# Biochemical Changes in Two Barley Genotypes Inoculated With a Beneficial Fungus *Trichoderma harzianum* Rifai T-22 Grown in Saline Soil

**DOI:** 10.3389/fpls.2022.908853

**Published:** 2022-08-02

**Authors:** Sneha Vinay Kumar Gupta, Penelope M. C. Smith, Siria H. A. Natera, Ute Roessner

**Affiliations:** ^1^School of BioSciences, University of Melbourne, Parkville, VIC, Australia; ^2^School of Life Sciences, La Trobe University, Bundoora, VIC, Australia; ^3^Metabolomics Australia, The University of Melbourne, Parkville, VIC, Australia

**Keywords:** salt, barley, *Trichoderma*, abiotic stress, metabolites, beneficial interactions, lipids

## Abstract

One of the most important environmental factors impacting crop plant productivity is soil salinity. Fungal endophytes have been characterised as biocontrol agents that help in plant productivity and induce resistance responses to several abiotic stresses, including salinity. In the salt-tolerant cereal crop barley (*Hordeum vulgare* L.), there is limited information about the metabolites and lipids that change in response to inoculation with fungal endophytes in saline conditions. In this study, gas chromatography coupled to mass spectrometry (GC–MS) and LC–electrospray ionisation (ESI)–quadrupole–quadrupole time of flight (QqTOF)-MS were used to determine the metabolite and lipid changes in two fungal inoculated barley genotypes with differing tolerance levels to saline conditions. The more salt-tolerant cultivar was Vlamingh and less salt tolerant was Gairdner. *Trichoderma harzianum* strain T-22 was used to treat these plants grown in soil under control and saline (200 mM NaCl) conditions. For both genotypes, fungus-colonised plants exposed to NaCl had greater root and shoot biomass, and better chlorophyll content than non-colonised plants, with colonised-Vlamingh performing better than uninoculated control plants. The metabolome dataset using GC–MS consisted of a total of 93 metabolites of which 74 were identified in roots of both barley genotypes as organic acids, sugars, sugar acids, sugar alcohols, amino acids, amines, and a small number of fatty acids. LC-QqTOF-MS analysis resulted in the detection of 186 lipid molecular species, classified into three major lipid classes—glycerophospholipids, glycerolipids, and sphingolipids, from roots of both genotypes. In Cultivar Vlamingh both metabolites and lipids increased with fungus and salt treatment while in Gairdner they decreased. The results from this study suggest that the metabolic pathways by which the fungus imparts salt tolerance is different for the different genotypes.

## Introduction

Soil salinity is one of the most common global issues that has a detrimental impact on crop productivity. Plant growth and development are hindered by salinity due to water stress, cytotoxicity from excessive absorption of ions like sodium (Na^+^) and chloride (Cl^−^), and nutritional imbalance. Salinity impairs plant growth and development *via* water stress, cytotoxicity due to excessive uptake of ions such as sodium (Na^+^) and chloride (Cl^−^), and nutritional imbalance. In 2018, the United Nations Food and Agricultural Organization (FAO; Food and Agriculture Organization of the United Nations, 2018) recognised this issue as a high priority which requires several approaches to reclaim and manage the affects and protect agricultural production. The challenge is worsened by the growing number of people to feed, with the world’s population expected to exceed 9 billion by 2050 and reach over 11 billion by 2,100 [United Nations Department of Economic Social Affairs (UN DESA), 2019].

In order to overcome agricultural productivity constraints, considerable amounts of agro-chemicals, such as synthetic fertilisers and pesticides, are utilised in many agricultural systems (Duan et al., 2016; Chowdhary et al., 2018). However, intensification of agriculture disrupts ecological balance, lowers soil fertility, contaminates the food chain, pollutes groundwater, diminishes microbial diversity, lowers soil pH, and increases microbial resistance (Uphoff and Dazzo, 2016). Therefore, sustainable methods are necessary to achieve increased agricultural productivity while maintaining ecological balance. This will improve plant resilience and the ability of plants to adjust to changing climatic circumstances, as well as biotic and abiotic stress, which will result in higher crop yields.

Use of microbial inoculants to enhance the availability and uptake of vital soil nutrients has a role in developing more sustainable and eco-friendly agricultural systems. Furthermore, these inoculants are particularly beneficial in reducing abiotic and biotic stresses in developing crops (Jambon et al., 2018). Several studies have highlighted the usefulness and benefits of microbe-based formulations in improving crop development and yield (Huang et al., 2014; Gangwar and Singh, 2018; Bulegon et al., 2019; Evelin et al., 2019).

In recent years, research has focused on the plant microbiome that colonises the internal tissues of the host plant without causing any disease symptoms. These are termed endophytes. Vertical seeding or horizontal transmission from the soil to the plants are both ways for endophytic microorganisms to infiltrate and colonise plants (Huang et al., 2016; Verma and White, 2018). This results in beneficial mutualistic interactions. Endophytes interact with their plant hosts, generate positive reactions in their host plants in response to abiotic environmental challenges, and synthesise essential bioactive metabolites (Khan et al., 2016). Several reports have described endophytic microorganisms which mediate beneficial traits/functions in their plant host (Huang et al., 2014; Gangwar and Singh, 2018; Bulegon et al., 2019; Evelin et al., 2019).

*Trichoderma* species are one group of fungal endophytes that impart beneficial effects on plants. They are widely distributed and can be found in soil, decomposing wood, and other fungi (Carreras-Villaseñor et al., 2012). Soil is an essential substrate for *Trichoderma*, and various researchers throughout the world have conducted investigations on soil-inhabiting species of the genus (Chen and Zhuang, 2017). *Trichoderma* have been long recognised as agents for the control of plant disease and for their ability to improve plant growth and development. However, recently it has become clearer that certain species also confer stress tolerance in plants (Velázquez-Robledo et al., 2011; Brotman et al., 2013; Contreras-Cornejo et al., 2014). For example, *Trichoderma* spp. imparted salt tolerance in wheat by increased accumulation of proline (Rawat et al., 2011). Microarray analysis of Arabidopsis and cucumber roots inoculated with *Trichoderma* spp. and exposed to salt stress indicated enhanced expression of genes linked to salt tolerance, osmoprotection mechanisms, and ascorbic acid (AA) synthesis (Brotman et al., 2013).

Gupta et al. (2021) showed that several metabolites and lipids are correlated with the positive effects of *T. harzianum* strain T-22 inoculation in roots of two barley genotypes under saline conditions when grown on agar medium. Here we have extended the study to look at a more natural medium for *Trichoderma* infection of barley to determine whether the *Trichoderma* changes the same metabolites in barley to impart salt tolerance. Therefore, for this study, the role of *Trichoderma* strain T-22 on two barley genotypes (Vlamingh and Gairdner) grown in saline soil was examined. The favourable effect of this fungus on both genotypes under control and saline conditions, as determined by numerous physiological parameters, is clearly seen in the biomass findings. Further, to enhance our knowledge following endophyte inoculation that are involved in conferring positive effects on barley plants grown on agar medium, the levels of metabolite and lipid impacted in roots grown in saline soil were measured. To examine metabolites and lipids in inoculated and uninoculated roots of both genotypes under control and saline conditions, we used gas chromatography and liquid chromatography both coupled to mass spectrometry (GC–MS and LC–MS).

## Materials and Methods

### Plant Material and Growth Conditions

Based on their known differences in germination phenology and salinity tolerance, the study selected two barley genotypes: Vlamingh as a salt tolerant cultivar and Gairdner as a salt sensitive cultivar (Gupta et al., 2019). In a previous study, cultivar Vlamingh seeds had a 95% germination rate after 4 days treatment with 200 mM NaCl, whereas for Gairdner, the germination rate was 75% (Gupta et al., 2019). In a glasshouse study (data not shown) comparing control and saline conditions (200 mM NaCl), Vlamingh had higher biomass production than other tested genotypes and Gairdner the lowest. All seeds were sourced from the University of Adelaide, Australia.

### Fungal Isolates and Growth Conditions

The American Type Culture Collection (ATCC, Washington, DC) provided a freeze-dried version of the endophyte *T. harzianum* Rifai strain T-22 (ATCC 20847). The pellet obtained in the ampoule was suspended in sterile distilled water and left undisturbed overnight at room temperature (25°C). The suspension was mixed well on the following day and inoculated on Potato Dextrose Agar (39 g/l PDA powder in de-ionised water) in solid medium on a petri plate. The plates were incubated in the dark for 5 days at 25°C before being exposed to light for 3 days. After 8 days of incubation, conidia began to grow. Before starting the symbiotic interaction, the plates were kept at room temperature.

### Material and Chemicals for Mass Spectrometric Analysis

Solvents and reagents of analytical or mass spectrometric grade were bought from Merck Millipore (Bayswater, VIC, Australia). A Synergy UV Millipore System (Millipore, United States) provided 18.2 Ω deionised water.

### Plant Growth Conditions and Inoculation With *Trichoderma harzianum*

To initiate fungal association with barley roots, seeds from both genotypes were sterilised by immersing them in 70% ethanol for 1 min and rinsing them 4–5 times in sterile 18.2 Ω deionised water, then immersing themin 1.0% (v/v) household bleach for 10 min and rinsing them thoroughly in sterile 18.2 Ω deionised water 6–7 times. Seeds were then imbibed overnight (∼16 h) in sterile 18.2 Ω deionised water with constant aeration. Sterilised seeds were transferred to cylindrical PVC plastic pipes filled with a sandy loam soil (Fultons, Australia) that had been pasteurised by steam for 1 h at 85°C and rapidly cooled by blowing air through the mix. The cylindrical plastic pipes were placed in a growth cabinet maintained at 17°C constant temperature with no light (Supplementary Figure 1) to allow germination of the sown seeds. At the same time as seeds were being germinated, a fresh conidial suspension was prepared by flooding a PDA plate containing visible conidia, with sterile 18.2 Ω deionised water, the subsequent suspension was collected and passed through a filter to separate conidia from hyphae. The suspension was diluted to yield 1 × 10^8^ CFU mL^−1^ conidia.

After 48 h, the conditions of the growth cabinet were adjusted to 17°C for 16 h light and 10°C for 8 h dark cycles for the remainder of the experiment. The average water holding capacity of soil in each pot was 300 ml, with approximately 20–30 ml water loss per day, whether taken up by the plant or evaporated to the environment. Prior to salt treatment, all plants were given an equal amount of water (200 ml) every alternate day.

After 7 d, 24 seedlings from each genotype were inoculated with a conidial suspension of 25 ml of 1 × 10^8^ CFU g^−1^ and 24 seedlings from each genotype received an equal amount of sterile 18.2 Ω deionised water. 7 d post inoculation, salt treated seedlings received 50 mM NaCl every 4 h in 50 ml of sterile 18.2 Ω deionised water (to prevent salt precipitation) until the NaCl concentration in each pipe reached 200 mM NaCl. A total of 12 replicates were allocated for each condition and each condition was assigned as follows; 0 mM NaCl (control) abbreviated as ‘C’, control with inoculum abbreviated as ‘*CF*’, 200 mM NaCl (salt) abbreviated as ‘S’, and salt with inoculum abbreviated as ‘SF’. Plants were harvested 48 h after the concentration of NaCl in the pipes reached 200 mM (Supplementary Figure 1).

### Shoot and Root Length, Weight and Chlorophyll Content Determination

The roots from six harvested plants from each condition and genotype were immersed in water and scanned using a flatbed scanner followed by analysis using WinRHIZO software (Regent Instruments, Canada) to determine total root length (TRL). Shoot lengths were determined at harvest by measuring above ground shoot length with a ruler. The aerial parts of the plants were separated and dried in an oven (75°C) for 48 h and weighed using a weighing scale. The chlorophyll content was measured as a spad value (SPAD-502Plus, United States). The remaining six plants of each treatment were harvested, and roots were immediately frozen in liquid nitrogen and stored in a − 80°C freezer until they were extracted metabolomics analyses.

### GC–MS Untargeted Analysis for Polar Metabolites

Fifty (50 ± 2) mg of fresh frozen root tissue harvested from both varieties of barley was extracted in 500 μl of 100% MeOH (Scharlau, city, country), containing 4% v/v of [^13^C_6_] Sorbitol/[^13^C_5_^15^N] Valine (0.5 mg mL^−1^). The samples were prepared according to the instructions in Gupta et al. (2021). Pooled blank quality control (PBQC) samples were prepared by combining 50 μl aliquots from each sample solution into a single sample. A GC–MS system comprising of a Gerstel 2.5.2 autosampler, a 7890A Agilent gas chromatograph, and a 5975C Agilent quadrupole mass spectrometer (Agilent, Santa Clara, United States) with an electron impact (EI) ion source was used to analyse samples with an injection volume of 1 μl for each derivatized sample. Instrumental settings were adapted from Dias et al. (2015). Calibration of polar metabolites was done using serial concentrations of calibration authentic standards. Detailed instrumental parameters are provided in Supporting Experimental Materials and Methods.

### LC–MS Untargeted Analysis for Lipid Analysis

LC–MS/MS was used to extract and analyse lipids, with modified procedures from Shiva et al. (2018) and Yu et al. (2018). Details are provided in Supporting Experimental Materials and Methods.

### Data Preprocessing and Statistical Analysis

The Automated Mass Spectral Deconvolution and Identification System (AMDIS) software [National Institute of Standards and Technology (NIST), Gaithersburg, MD, United States] was used to perform *in silico* data pre-processing for mass spectral deconvolution, peak picking, and identification for GC–MS data analysis. The PBQC file was used to create a target component library (TCL).

Peak identification was confirmed using Agilent MassHunter Qualitative Analysis B.05.00 (Agilent Technologies, Inc. 2011). Batch and method files were created with Agilent MassHunter Quantitative Analysis software (for GC–MS) Version B.07.00/Build 7.0.457.0 (Agilent Technologies, Inc. 2008) and then exported to Microsoft Excel. Manual integration was done to correct for baseline drift using the former software. The exported peak area data from the batch file was normalised to internal standard [^13^C_6_] sorbitol, and fresh weight of each root sample. Data was also log transformed, and Student’s t-test *p*-values were calculated. The p-values were further subjected to Benjamini-Hochberg False Discovery Correction (Benjamini and Hochberg, 1995).

The six biological replicates were then subjected to multivariate data analysis using MetaboAnalyst 3.0 (Xia et al., 2015) software. Unsupervised learning algorithms such as principle component analysis (PCA) and hierarchical cluster analysis were used to determine pattern recognition (HCA). Log data transformation and mean centering were applied for the PCA. Ward’s clustering was used in a hierarchical cluster analysis utilising Euclidean distances. Further, the construction, interaction, and pathway analysis of significantly changed metabolites in both genotypes was performed with MetaboAnalyst 3.0 (https://www.metaboanalyst.ca/MetaboAnalyst/upload/PathUploadView.xhtml) based on database sources including KEGG (http://www.genome.jp/kegg/) to identify the top altered pathways analysis and for visualisation. GraphPad Prism 8.0 (GraphPad Software, Inc., United States; Full Version) was used to create fold-change for univariate analysis and bar charts.

For LC–MS data analysis, raw HPLC-MS data was visually inspected for integrity using PeakView Sciex Software (ver. 2.2), and raw LC-TripleTOF-MS data containing m/z RT (mass to charge_ retention time) and associated peak intensities were converted to ABF (analysis base file) format using the Reifycs file converter before being processed using MS-DIAL 3.98. (http://prime.psc.riken.jp/Metabolomics_Software/MS-DIAL/index2.html, accessed 18 September 2019; Tsugawa et al., 2015). Statistical analysis was performed using MetaboAnalyst 3.0 (http://www.metaboanalyst.ca/, accessed 10 September 2019). MS-DIAL export was done using the default parameters for a Lipidomics ‘omics project. After manually inspecting the raw data, the MS peak filter threshold was established. In addition, the adduct ion search was set to look for ([M + H]+), ([M + Na]+), ([M + K]+), and ([M + NH_4_]+) adducts, with a retention time tolerance of ±0.5 min and a MS1 tolerance of 0.1 Da. A csv file was created from a matrix including tentatively identified m/z RTs and their associated intensity peaks. The Metabolomics Standards Initiative standards were used to determine the level of identification (Sumner et al., 2007). Absolute quantities were determined, and the concentration unit was expressed as picomole/mg of fresh weight.

Multiple comparison statistical analyses were performed on the processed lipid data using Analysis of Variance (ANOVA) with a false discovery rate (FDR)-adjusted *p* value of 0.05 and the Benjamini–Hochberg technique. MetaboAnalyst was used to develop a list of features (m/z_RT) with a false discovery rate corrected value of *p* (*p <* 0.01) fold change of two or more in peak intensity relative to the respective controls using additional statistical analysis (Student’s t-tests) comparing each treatment in both genotypes. All bar charts were generated using GraphPad Prism 8.0 (GraphPad Software, Inc., United States; Full Version).

## Results

### Effect of Fungus and Salt Stress on Seminal Root Length of Vlamingh and Gairdner

**Figure 1:**
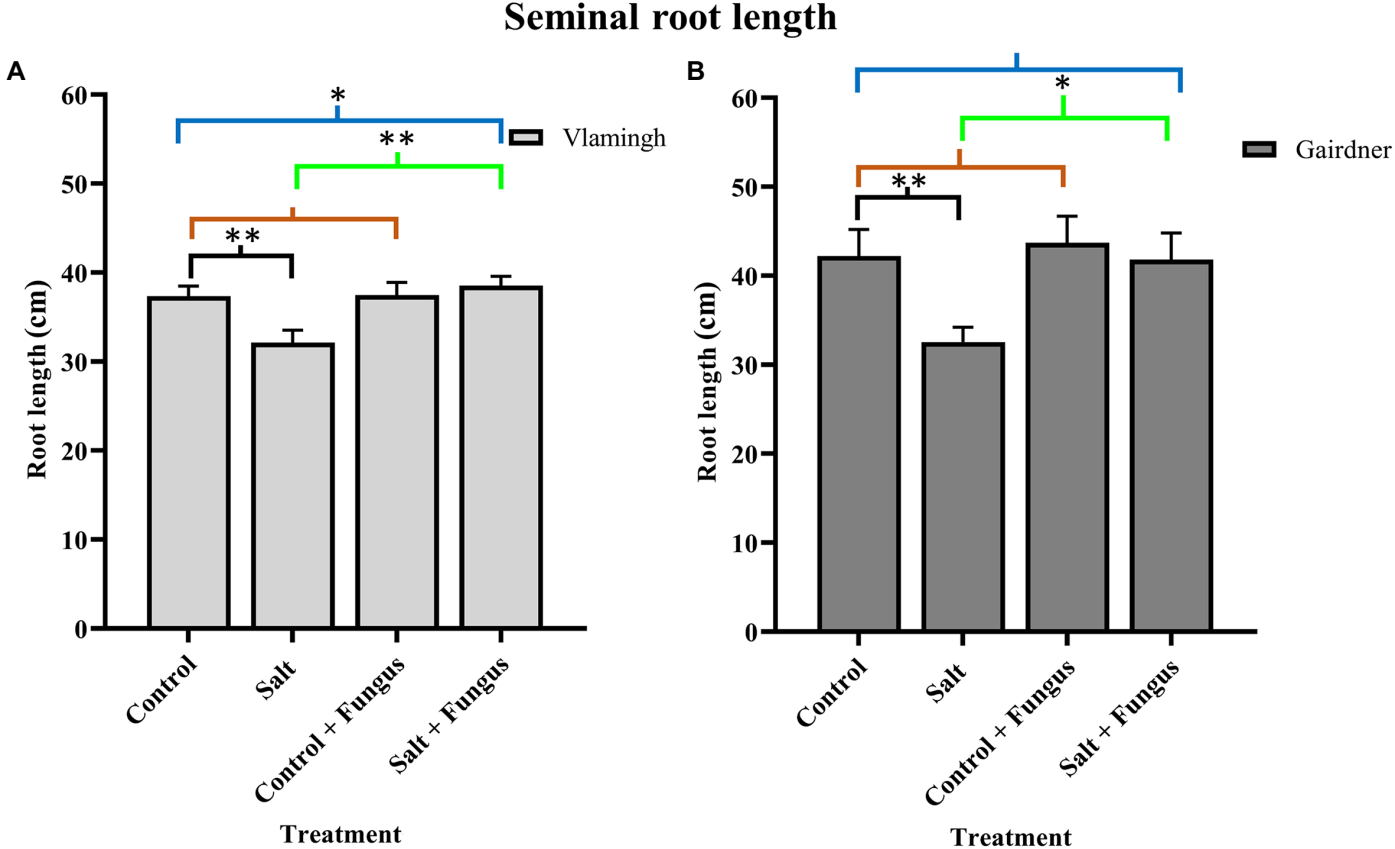
Seminal root length of barley roots of two genotypes, cv. Vlamingh **(A)** and cv. Gairdner **(B)** under four treatment conditions for seedlings grown in soil. X axis represent treatments and Y axis represent lengths in cm. *n* = 6 for both genotypes. Different comparisons are represented by three colour bars above the graphs: black represents comparisons between salt and control, light brown represents comparisons between control + fungus and control, and cyan green represents comparisons between salt + fungus and salt. As established by Student’s t-test, asterisks imply significance for *P <* 0.05 (one asterisk) and *p <* 0.01 (two asterisks).

Nodal roots could not be distinguished from seminal roots due to soil aggregates in both genotypes. Therefore, average total seminal root length (SRL) was measured. For phenotyping seedlings, this method of measuring provided a relatively quick and accurate alternative. When grown in control conditions, the sensitive genotype Gairdner had higher SRL than the tolerant genotype Vlamingh. Although SRL was increased in inoculated seedlings not treated with salt (*CF*) compared to control seedlings (C) in both genotypes, the increase was greater in Gairdner (3.50%) than in Vlamingh (0.35%; Figure 1; Table 1).

**Table 1:** Percentage increase in seminal root length in two barley genotypes, cv. Vlamingh and cv. Gairdner.

Treatments	Increase in SRL (%)	
	Vlamingh	Gairdner
*CF* vs. C	0.35	3.51
SF vs. S	19.95	28.48
SF vs. C	3.21	−0.95

The SRL was lowered by salt treatment of uninoculated plants (S) for both genotypes, although the reduction was more pronounced for Gairdner (Figure 1). In inoculated plants grown without salt (*CF*), SRL increased in both genotypes, but the increase was more marked for Gairdner as shown in Figure 1. In the inoculated salt treatment (SF), SRL increased significantly by 20.0% (*p <* 0.01) in Vlamingh and 28.50% (*p <* 0.05) in Gairdner salt (SF) compared to uninoculated plants (S). The SRL in seedlings grown with fungus and treated with salt (SF) was larger than that of control (C) seedlings in Vlamingh (3.21% (*p <* 0.05)). However, the SRL for Gairdner seedlings grown with fungus and treated with salt was slightly reduced as compared to control seedlings (−0.9%).

### Root and Shoot Dry Weight

**Figure 2:**
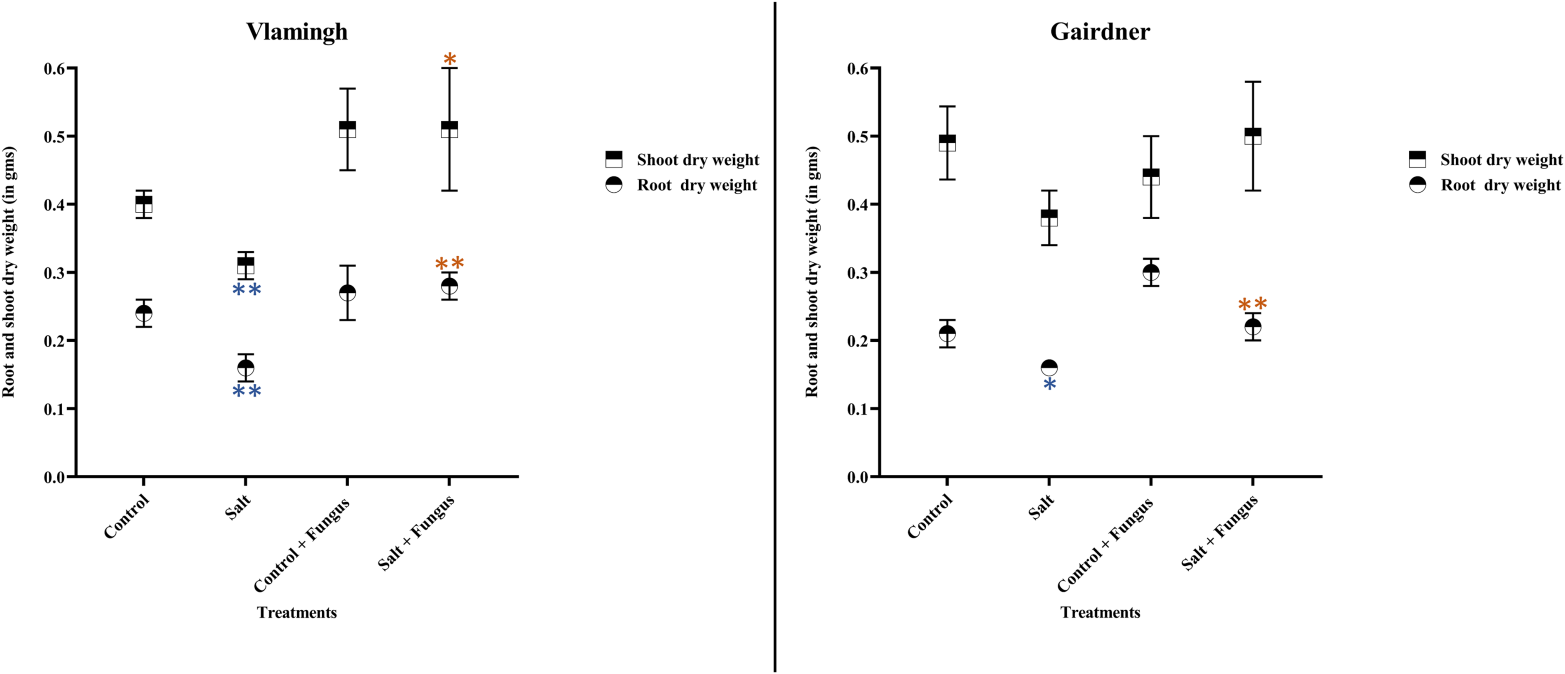
Averages and std. errors of showing dry weight of roots and shoots of barley genotypes Vlamingh and Gairdner under four treatment conditions. X axis represent the four treatment conditions and Y axis represent weights in grams. *n* = 6 for both genotypes. As established by Student’s t-test, asterisks imply significance for *p <* 0.05 (one asterisk) and *p <* 0.01 (two asterisks). Three comparisons were made-uninoculated salt treatment vs. uninoculated control treatment, fungus inoculated control treatment vs. uninoculated control treatment and fungus inoculated salt treatment vs. uninoculated salt treatment. Blue colour indicates significant reduction and orange colour indicates significant increase.

Root dry weight in uninoculated plants treated with salt (S) was reduced significantly in both genotypes when compared to the same cultivar grown without salt (C; Table 2; Figure 2). No significant changes were observed when inoculated plants grown without salt (*CF*) were compared to uninoculated plants grown without salt (C). In the inoculated salt treatment (SF), root dry weight increased in both genotypes compared to treatment S (no inoculation).

**Table 2:** Physiological parameters measured in cv. Vlamingh and cv. Gairdner under four treatment conditions.

	Vlamingh				Gairdner			
Measurements	Uninoculated control treatment	Uninoculated salt treatment	Fungus inoculated control treatment	Fungus inoculated salt treatment	Uninoculated control treatment	Uninoculated salt treatment	Fungus inoculated control treatment	Fungus inoculated salt treatment
Root fresh weight (g/plant)	0.36 ± 0.02	0.32 ± 0.01	0.35 ± 0.3	0.35 ± 0.3	0.36 ± 0.04	0.38 ± 0.03^**^	0.45 ± 0.05	0.3 ± 0.02^*^
Root dry weight (g/plant)	0.24 ± 0.02	0.16 ± 0.02^**^	0.27 ± 0.04	0.28 ± 0.02^**^	0.21 ± 0.02	0.16 ± 0.01^*^	0.3 ± 0.02	0.22 ± 0.02^**^
Shoot fresh weight (g/plant)	0.69 ± 0.05	0.5 ± 0.04^**^	0.82 ± 0.03	0.74 ± 0.06^*^	0.76 ± 0.05	0.66 ± 0.01	0.74 ± 0.05	0.7 ± 0.09
Shoot dry weight (g/plant)	0.4 ± 0.05	0.31 ± 0.04^**^	0.51 ± 0.05	0.51 ± 0.05^*^	0.49 ± 0.02	0.38 ± 0.02	0.44 ± 0.06	0.5 ± 0.09
Root: shoot ratio	0.6	0.52	0.53	0.55	0.43	0.42	0.68	0.44

Root and shoot fresh weight were measured immediately after harvest and dry weights of both were measured after drying in oven at 75°C for 48 h. *n* = 6 for all treatments and results are presented as mean ± standard error for all except root:shoot ratio. Asterisks denote significance for *p* < 0.05 (one asterisk) and *p* < 0.01 (two asterisks) as determined by Student’s t-test. Three comparisons were made-uninoculated salt treatment vs uninoculated control treatment, fungus inoculated control treatment vs uninoculated control treatment and fungus inoculated salt treatment vs uninoculated salt treatment.

In comparison to the same cultivar grown without salt (C), salt treatment of uninoculated plants (S) reduced shoot dry weight in both genotypes, but the reduction was more pronounced for Vlamingh (Table 2; Figure 2). When comparing inoculated plants grown without salt (*CF*) to the same cultivar grown without salt and fungus (C), shoot dry weight increased in Vlamingh but decreased in Gairdner. In the inoculated salt treatment (SF), an increase in shoot dry weight was observed in Vlamingh compared to treatment S (no inoculation) and though there was an increase in Gairdner, it was not statistically significant due to high variation between samples.

### Root to Shoot Biomass Ratio

The root to shoot biomass ratio was measured in all treatments of both genotypes (Table 2). Under salt stress in uninoculated plants, the root to shoot biomass ratio decreased in both genotypes compared to those grown without salt, but the reduction was comparatively higher in Vlamingh (Table 2). In inoculated plants grown without salt (*CF*), root to shoot biomass ratio was reduced in Vlamingh (0.53) and increased in Gairdner (0.68) when compared to uninoculated seedlings (Vlamingh [0.6], Gairdner [0.43]). Inoculation with fungus under salt (SF) increased root to shoot biomass ratio by 0.03 in Vlamingh and 0.02 in Gairdner compared to uninoculated salt treated seedlings.

### Chlorophyll Content

**Figure 3:**
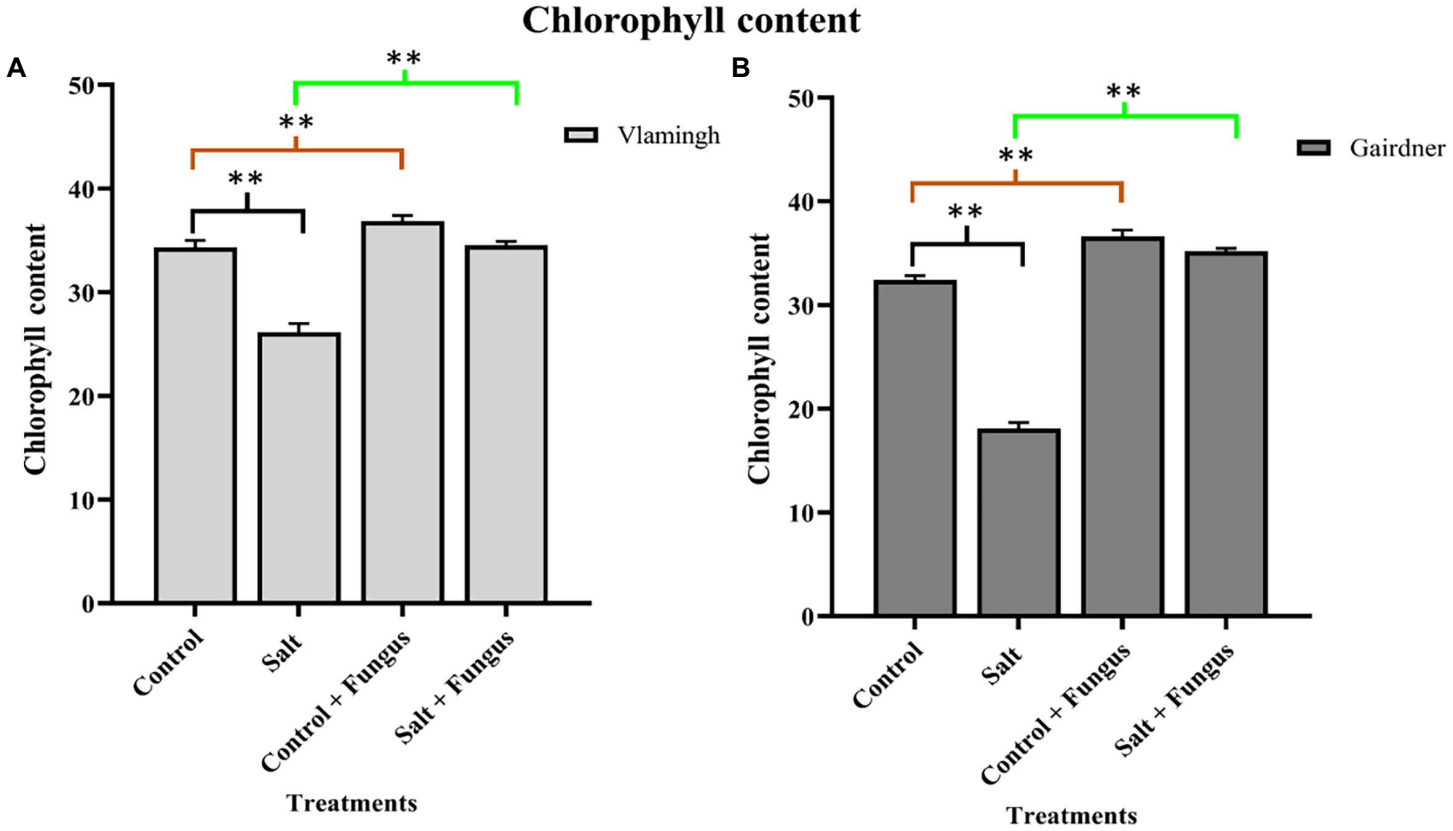
Leaf chlorophyll content of two barley genotypes cv. Vlamingh **(A)** and cv. Gairdner **(B)** measured using SPAD metre. The four treatments are represented on the X axis, and the chlorophyll index is represented on the Y axis. Different comparisons are represented by three colour bars above the graphs: black represents comparisons between salt and control, light brown represents comparisons between control + fungus and control, and cyan green represents comparisons between salt + fungus and salt. As determined by Student’s t-test, asterisks imply significance for *p <* 0.01 (two asterisks).

Leaf chlorophyll content of plants was measured to examine the effect of fungal inoculation with *T. harzianum* T-22 and/or salt treatment on the photosynthetic potential in both genotypes (Figure 3). In both genotypes, salt treatment of uninoculated plants (S) reduced chlorophyll content, although the difference was higher in Gairdner (Figure 3). The chlorophyll content increased in both genotypes in inoculated plants grown without salt (*CF*), and the impact was larger in Gairdner compared to uninoculated roots of the same cultivar (C). When seedlings were grown with the fungus and treated with salt (SF), the chlorophyll content was significantly increased in both genotypes compared to S making it equivalent to, or higher, than those for untreated control seedlings (C; Figure 3).

### The Effect of Inoculation With *Trichoderma harzianum* and Salt Stress on Polar Metabolites in Roots

A total of 93 metabolites, of which 75 were identified, were detected in roots of both barley genotypes using GC–MS. The identified metabolites were categorised according to compound classes and are listed in Supplementary Table S1. Among the identified metabolites, 27 were organic acids, 23 were sugars or sugar derivatives, 18 were amino acids and seven were fatty acids. Unknowns were categorised as compounds that did not return a match to the mass spectral library. The average normalised responses of all metabolites in all treated barley roots can be found in Supplementary Table S2.

**Figure 4:**
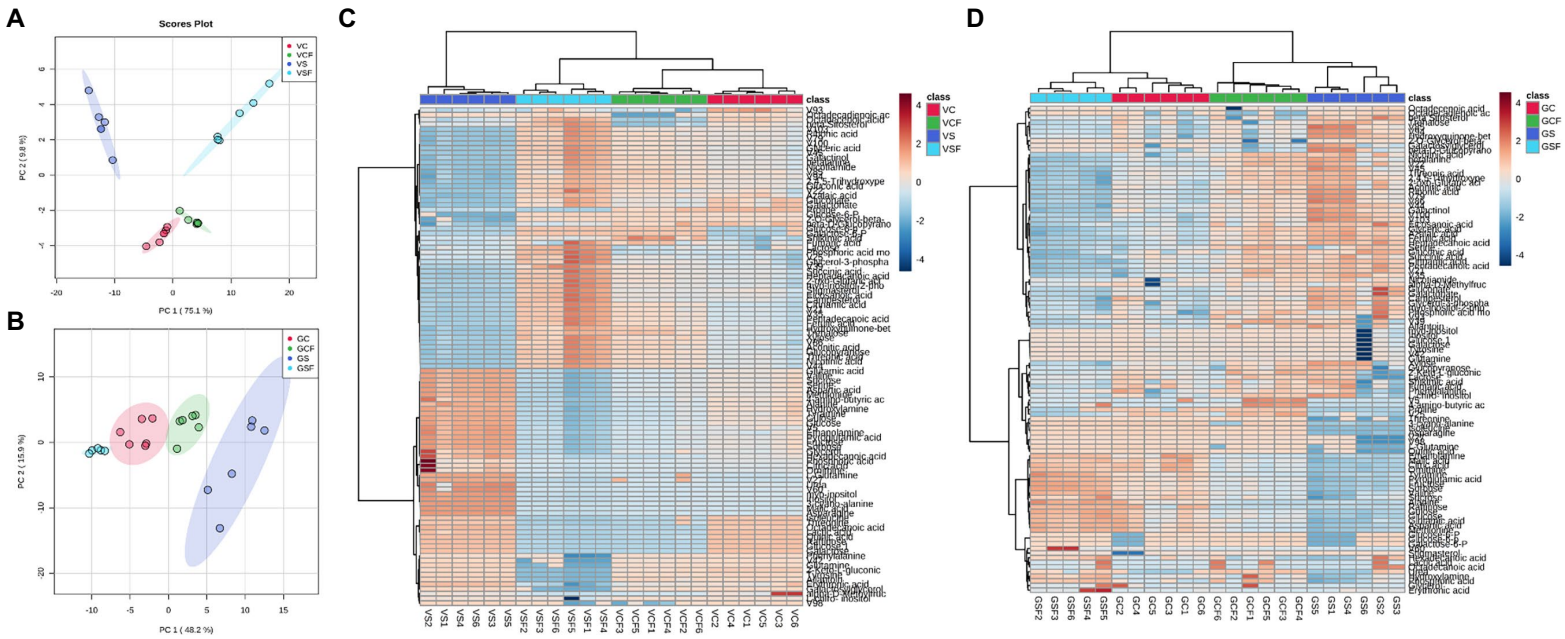
Principal Component Analysis (PCA) score plot and Heatmaps for cv. Vlamingh **(A,C)** and cv. Gairdner **(B,D)** under four treatments—C, control treated roots, S, Salt treated roots (200 mM NaCl), *CF*, control + fungus, SF, salt + fungus. The fungal inoculated samples (VCF and VSF) separate from the uninoculated samples (VC and *VS*) in cv. Vlamingh on component 1 (PC1). The salt treatment contributed to distinct separation on PC2 in cv. Vlamingh. In cv. Gairdner, PC1 clearly separates treatment GS from the other three treatments (GCF, GSF and GC). Unsupervised hierarchical clustering of lipid species is represented by the heatmaps (rows). The samples are represented in the column, while the detected lipid species are displayed in the row. Metabolites with a lower relative abundance are shown in blue, whereas those with a higher relative abundance are shown in red. The R package heatmap was used to cluster the data using Euclidean distance matrices.

Data dimension reduction performed by principal component analysis (PCA) plots for pooled biological quality control (PBQC) samples showed clear clustering between replicates from all treatments (Supplementary Figure 2). This indicates good reproducibility and reliable detection by the method and therefore data can be used for subsequent analysis and interpretation. Further For both genotypes, PCA plots were created, which revealed a well-defined distinction among samples from the different treatments (C, *CF*, S, and SF; Figures 4A,B) In Vlamingh and Gairdner, the first and second principal components accounted for 84.9 percent and 64.1 percent of the variation, respectively.

In both genotypes, a heatmap depicting changing levels of metabolites under the four different treatment settings (Figures 4C,D) reveals a clear distinction between the treatments. The uninoculated salt treatment was separated from the other treatment groups in Vlamingh forming two significant clusters. Further clustering was identified, resulting in the formation of a subclass that grouped C and *CF* together, while treatment SF was clustered apart. This means that salt treatment had a greater impact on root metabolite profiles in Vlamingh than all other treatments. In Gairdner (Figure 4D), two major clusters grouped treatments SF and C together and treatments S and *CF* together. This implies fungal inoculation under salt stress reduced the changes in metabolite profiles in Gairdner so they were similar to those of uninoculated control roots whereas the salt stress caused greater changes in the metabolite profile of uninoculated roots.

**Figure 5:**
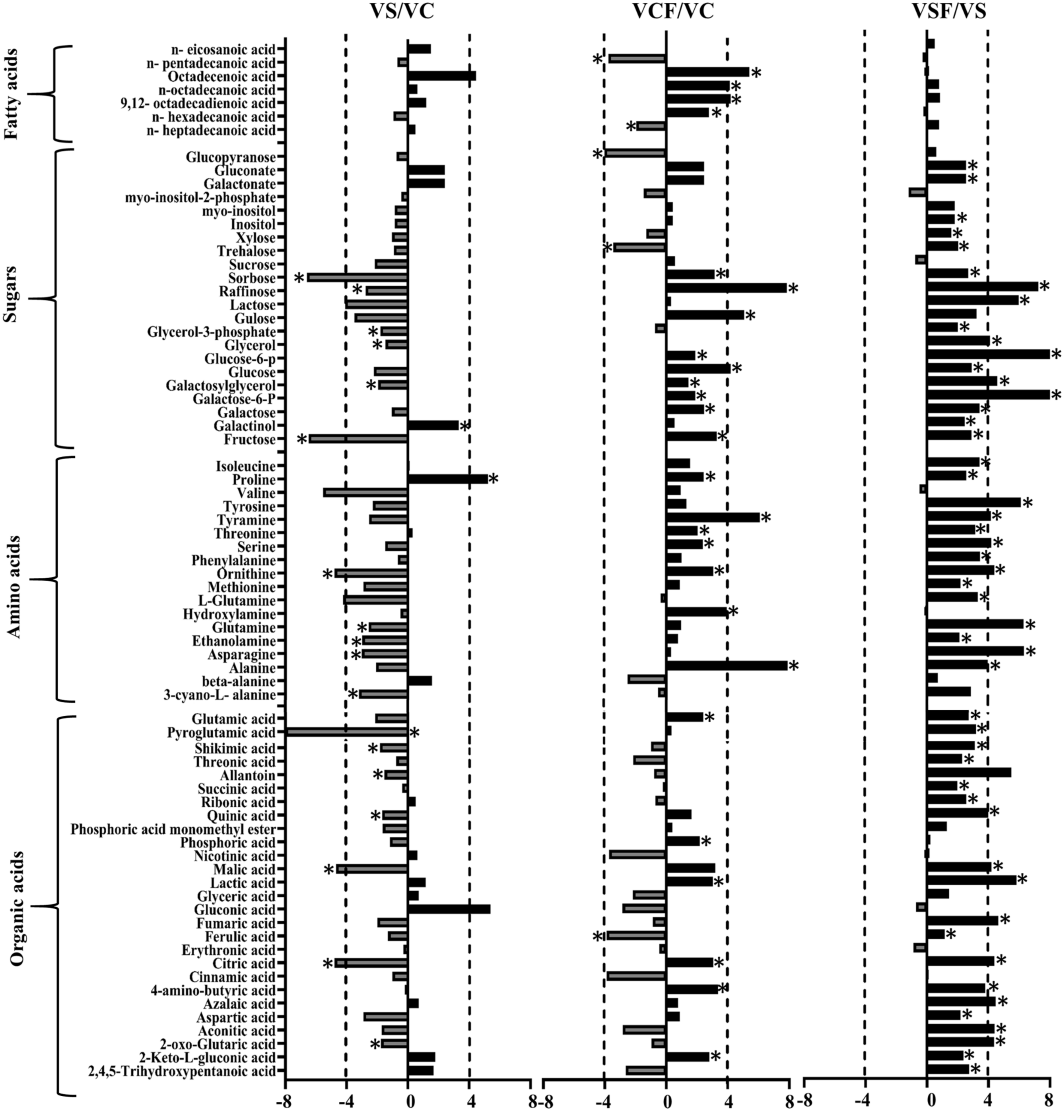
The graphs represent salt grown vs. control grown (*VS*/VC); control with fungus vs. control grown (VCF/VC); and salt with fungus vs. salt grown (VSF/*VS*) logarithmic ratios of representative sugars, organic acids, and amino acids in roots of barley cv. Vlamingh (V). An asterisk (^*^) indicates that the values are significantly different (*p <* 0.05, FDR). A dashed line indicates a four-fold change threshold.

**Figure 6:**
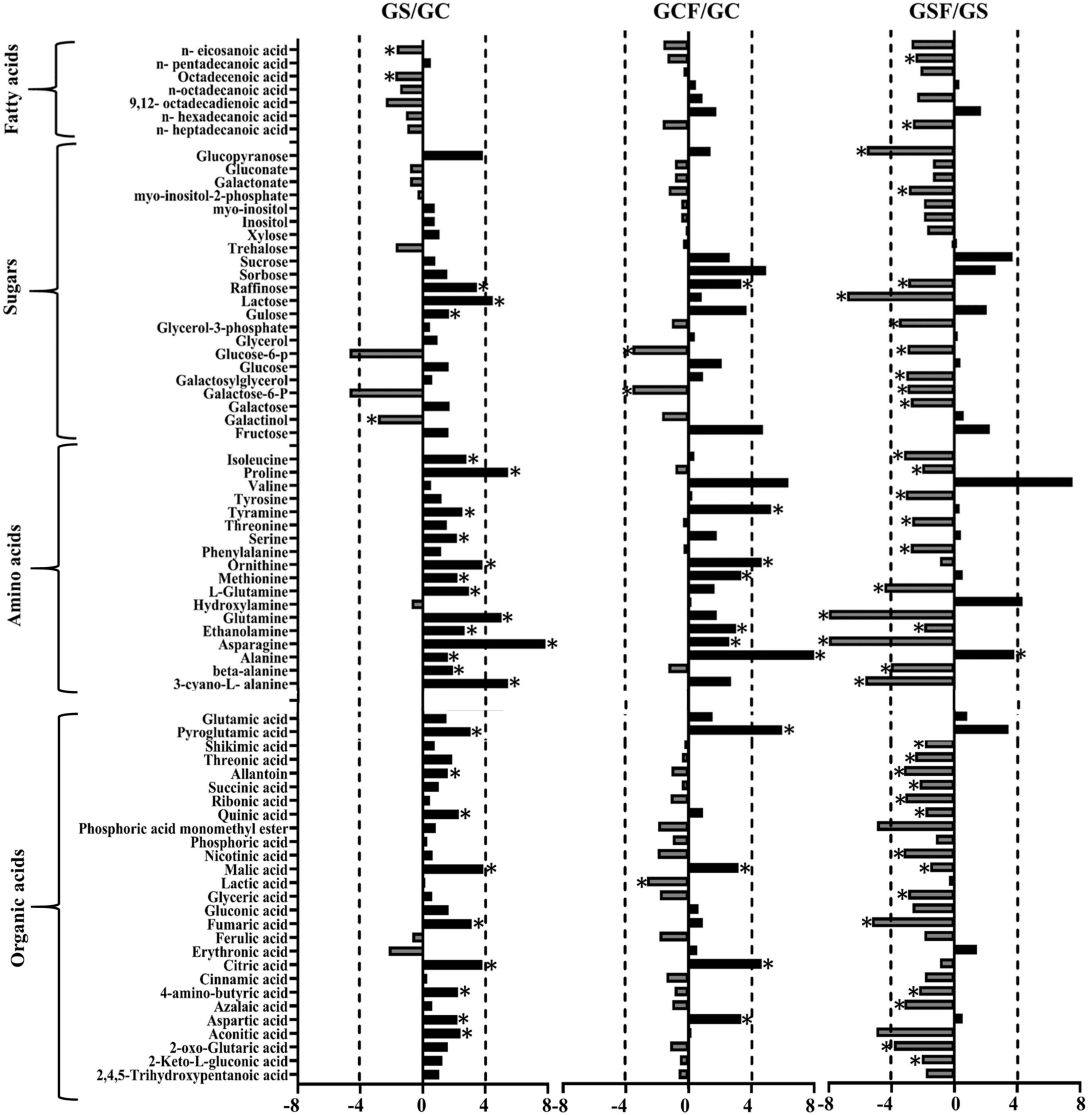
The graphs represent salt grown vs. control grown (GS/GC); control with fungus vs. control grown (GCF/GC); and salt with fungus vs. salt grown (GSF/GS) logarithmic ratios of representative sugars, organic acids, and amino acids in roots of barley cv. Gairdner (G). An asterisk (^*^) indicates that the values are significantly different (*p <* 0.05, FDR). A dashed line indicates a four-fold change threshold.

The fold change of metabolites between the different treatments in both genotypes was also analysed. Figures 5, 6 show a graphical representation of X-fold changes for treatment groups S and *CF* compared to treatment C. as well as SF compared to treatment S in Vlamingh and Gairdner, respectively.

When salt treatment was compared to control roots in both genotypes (Figures 5, 6), Vlamingh showed the greatest increases only in galactinol (+3.2-fold) and proline (+6.15-fold), whereas 14 out of 20 amino acids increased in Gairdner of which asparagine (+15.21-fold) showed the greatest increase. The greatest decrease after salt treatment was seen in pyroglutamic acid (−26.39-fold) in Vlamingh and galactinol (−3.33-fold) reduced greatly in Gairdner.

When control roots were inoculated with fungus in Vlamingh, the metabolites that increased most compared to uninoculated roots were raffinose (+15.19-fold), gulose (+5.80-fold) and alanine (+15.41-fold). However, in Gairdner inoculated roots, alanine (+30.22-fold), pyroglutamic acid (+7.88-fold), tyramine (+6.19-fold) and ornithine (+5.03-fold) showed the greatest increases compared to uninoculated roots. Few metabolites decreased in Vlamingh after fungal inoculation with glucopyranose (−2.02-fold) showing the greatest reduction compared to uninoculated roots. In Gairdner glucose-6-phosphate (−1.8-fold), galactose-6-phosphate (−1.8-fold) and lactic acid (−1.32-fold) were decreased when inoculated seedlings were compared to uninoculated roots.

When roots with fungal inoculation and treated with salt were compared to uninoculated roots only treated with salt, 17 of the 23 identified sugars increased significantly (*p <* 0.05) by +2-fold or greater in Vlamingh (Supplementary Table S1; Figure 5). Among 18 identified amino acids, 16 increased significantly (*p <* 0.05) showing greater than or equal to +2-fold, and 17 out of 27 organic acids increased significantly (*p <* 0.05) showing a + 2-fold or more increase. When Gairdner inoculated roots treated with salt were compared to uninoculated roots treated with salt, only asparagine (+8.88-fold) increased while 36 of the 75 identified metabolites showed reduced abundance (Supplementary Table S1).

To better understand the relevant pathways involved and affected, pathway analyses was performed using MetaboAnalyst. Supplementary Figure 3 shows an example of the comparison between the metabolome view with pathway impact on X-axis and log (*p* value) on Y-axis. Further, the corresponding ‘pathway view’ was used to visualise the pathways affected for a specific treatment comparison. For both genotypes, the top 10 pathways affected in all treatments were the same (Supplementary Figure 4).

### Untargeted Lipid Profiling of Barley Roots

An untargeted strategy was followed to extract the information from the large data sets obtained in LC-ESI-QqTOF-MS analyses in positive ionisation mode in both genotypes. A total of 3,645 m/z_RT features were detected (Supplementary Table S3) using MS-DIAL. PCA analysis was performed to identify the effect of all treatments in both genotypes. The first and second principal components accounted for 25.8% of the variation for both genotypes. Supplementary Figure 5a shows a separation of all treatments into two groups based on the genotypic variation. PC1 failed to show any clear clustering based on either treatments or genotypes. However, PC2 separates the two genotypes contributing to 11.2% of the variation. A hierarchical clustering analysis (HCA) heatmap showed a clear separation between two genotypes (Supplementary Figure 5b). No distinct further clustering was introduced by the inoculation of fungus in both genotypes. Therefore, untargeted analyses showed clustering of the mass features predominantly based on the barley genotype demonstrating a strong genetic impact on the root lipidome.

### Targeted MS/MS Lipid Analysis

By comparing retention time, precursor m/z, isotopic ratios, and MS/MS spectrum, MS-in DIAL’s silico LipidBlast database and data from Kehelpannala et al. (2020) were utilised to identify lipids. Peak identifications for false positives and true positives were manually checked, curated, and modified as needed. Of 3,645 m/z_RT features, 186 lipid molecular species were identified with MS/MS spectra and categorised into different lipid classes (Supplementary Table S4). Table 3 shows the number of lipid species in each class and the total percentage of individual species detected in each lipid class from roots of both genotypes. Further analyses were performed on the identified lipids in both genotypes and the following sections will only discuss the significant changes in identified lipids. An unsupervised chemometric analysis of the identified lipids datasets by principal component analysis (PCA) separated treatment SF of Vlamingh from the other groups along PC1 representing 43.9% of the variation (Supplementary Figure 7). This indicates a substantial difference in the lipidome of inoculated roots of Vlamingh under salt stress. A similar plot was observed when both genotypes were separately analysed using PCA as given in Supplementary Figure 8a.

**Table 3:** Number of detected lipid species in roots of two barley genotypes (cv. Vlamingh and cv. Gairdner) from four treatment groups: control C, control inoculated with fungus *CF*, salt (200 mM NaCl) S, and salt inoculated with fungus SF. An LC-TripleTOF-MS was used for the analysis (positive ionisation mode).

Lipid class	Lipid subclass	Species identified
Glycerophospholipids (GP) (total = 109)	PC	35
	LPC	23
	PE	23
	LPE	6
	PG	7
	PI	3
	PS	12
Glycerolipids (GL) (total = 65)	DG	24
	TG	41
Sphingolipids (SL)	CE	5
Fatty acyls (FA)	ACar	7

PC, phosphatidylcholine; LPC, lysophosphatidylcholine; PE, phosphatidylethanolamine; LPE, lysophosphatidylethanolamine; PG, phosphatidylglycerol; PI, phosphatidylinositol; PS, phosphatidylserine; DG, diacylglycerol; TG, triacylglycerol; CE, ceramides; and ACar, acyl carnitines.

Further univariate analysis was performed in roots of both genotypes to identify important lipids with a focus on those that changed due to (a) salt stress in both genotypes, (b) the interaction of fungus with roots under control conditions and (c) the effect of salt stress in roots inoculated with the fungus. Volcano plots were used (Supplementary Figure 9) to compare the size of the fold change (+1.5-fold or greater for this study) to the statistical significance level.

Table 4 gives the directionality of changes in lipid species as identified using volcano plots, in three treatment group comparisons in both genotypes. The largest number of lipid species that changed significantly in both genotypes were members of the Glycerolipids (GLs) and Glycerophospholipids (GPs) classes. Therefore, a more rigorous statistical analysis based on pairwise comparisons between the four treatments in both genotypes was carried out for GPs and GLs in order to adequately understand the directionality of the changes in the identified features. In the following paragraphs, lipid nomenclature follows the ‘Comprehensive Classification System for Lipids’ given by the International Lipid Classification and Nomenclature Committee (ILCNC). For example, PC (38:n) denotes a PC species having a fatty acyl sum composition of 38 carbons and all identified double bonds in this study, which is denoted by the letter ‘n’. Supplementary data contains information on the number of double bonds in each particular lipid species (Supplementary Table S5).

**Table 4:** Number of significantly altered lipid species (*p* < 0.05) with ≥1.5 fold-changes in relative abundance between the three treatment group comparisons for two barley genotypes.

		Number of lipid species increased or decreased by ≥1.5-fold	
Treatment specific		Vlamingh	Gairdner
Salt vs. Control	Higher	14	13
	Lower	15	1
Control + fungus vs. Control	Higher	47	1
	Lower	8	6
Salt + fungus vs. Salt	Higher	102	2
	Lower	0	8

**Figure 7:**
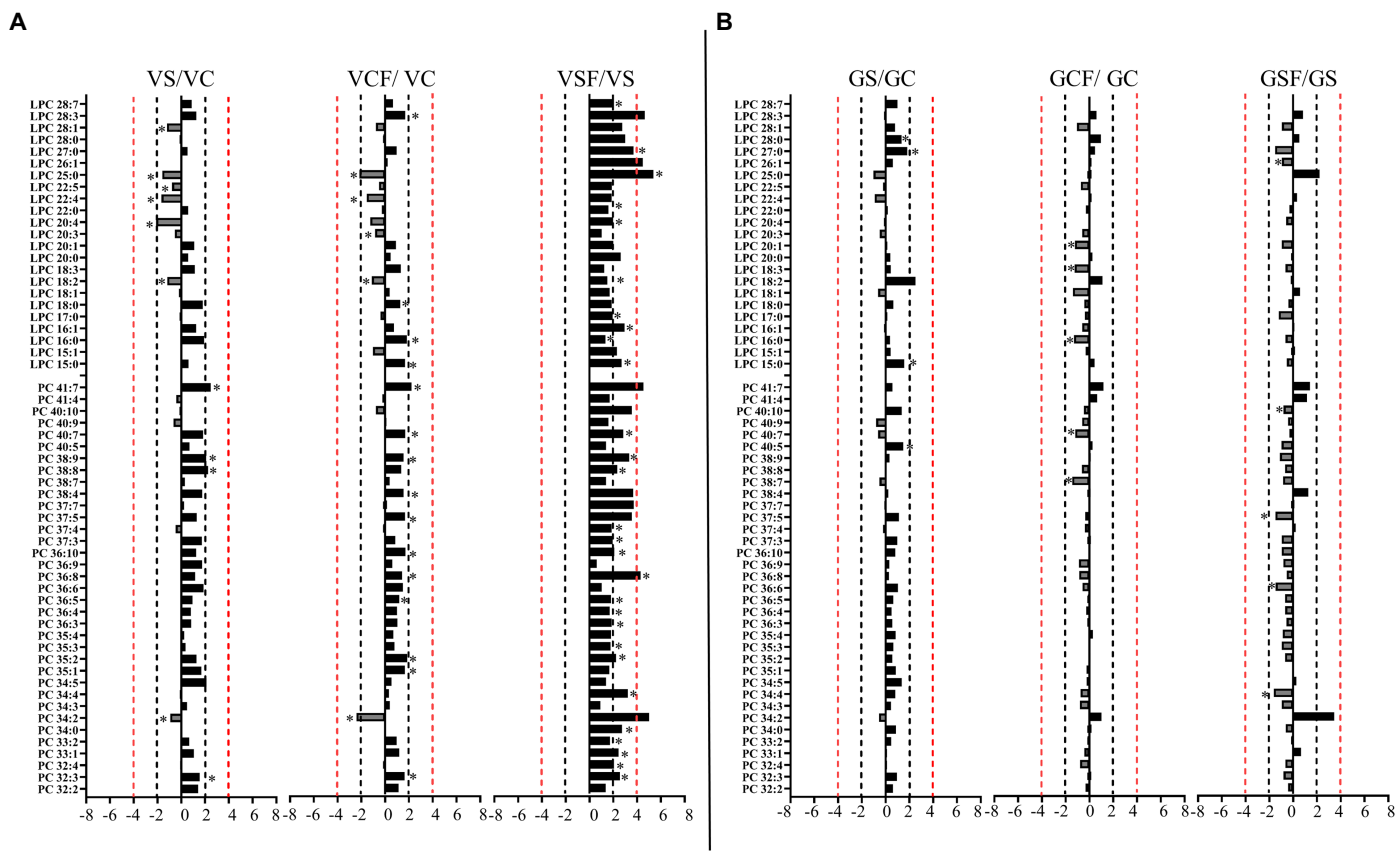
Logarithmic ratios of representative phosphatidylcholine (PC) and lysophosphatidylcholine (LPC) content in roots of barley cv. Vlamingh (V) given as **(A)** and cv. Gairdner (G) given as **(B)**, showing three comparisons—salt grown (200 mM NaCl) vs. control grown (*VS*/VC and GS/GC); control with fungus vs. control grown (VCF/VC and GCF/GC); salt and fungus grown vs. salt grown (VSF/*VS* and GSF/GS). An asterisk (^*^) indicates that the values are significantly different (*p <* 0.05, FDR). A black dashed line indicates a 2-fold change threshold, whereas a red dashed line indicates a 4-fold change threshold. All treatments included a total of six replicates (*n* = 6).

**Figure 8:**
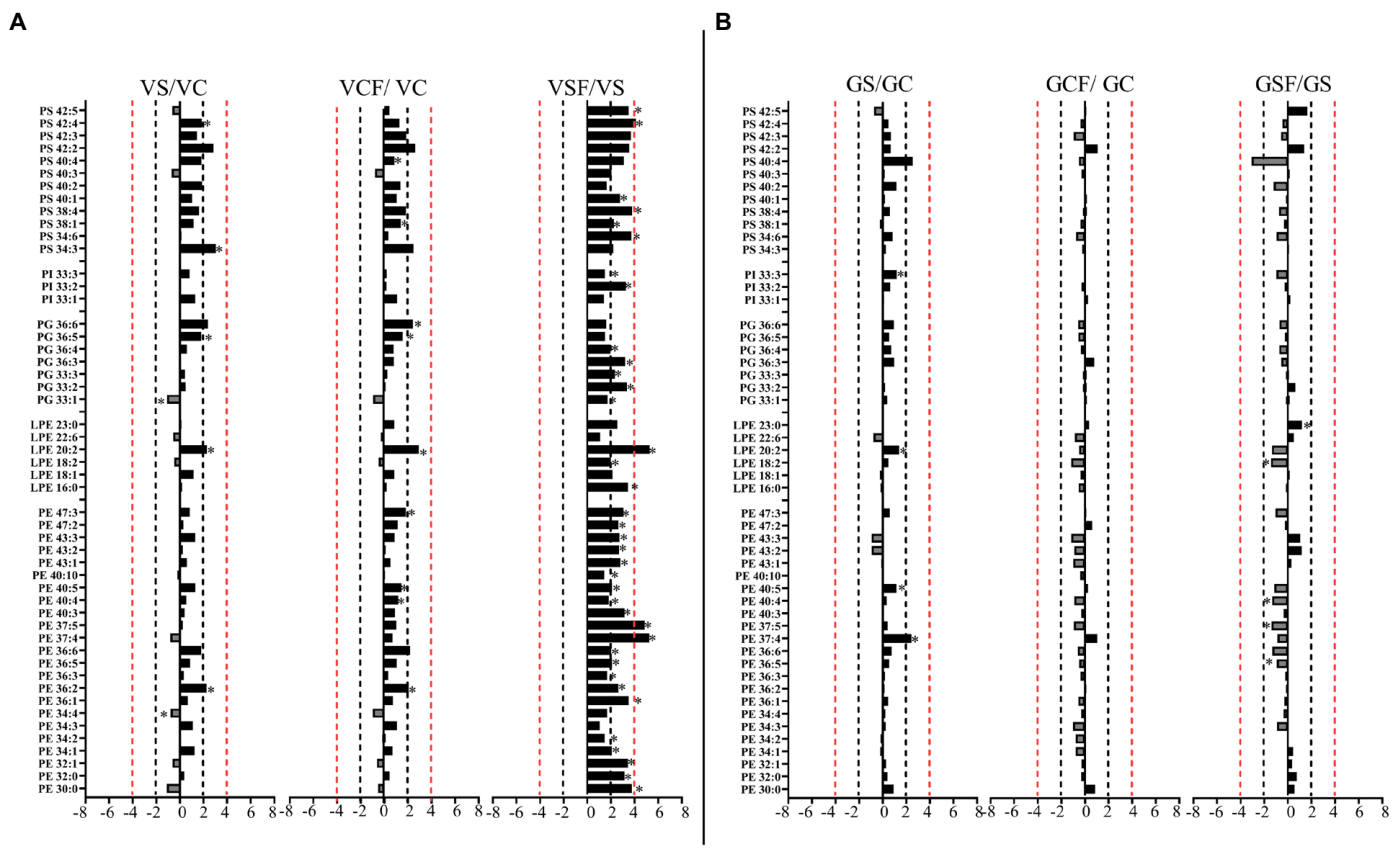
Logarithmic ratios of representative phosphatidylethanolamine (PE), lysophosphatidylethanolamine (LPE), phosphatidylglycerol (PG), phosphatidylinositol (PI) and phosphatidylserine (PS) content in roots of barley cv. Vlamingh (V) given as **(A)** and cv. Gairdner (G) given as **(B)**, showing three comparisons—salt grown (200 mM NaCl) vs. control grown (*VS*/VC and GS/GC); control with fungus vs. control grown (VCF/VC and GCF/GC); salt and fungus grown vs. salt grown (VSF/*VS* and GSF/GS). An asterisk (^*^) indicates that the values are significantly different (*p <* 0.05, FDR). A black dashed line indicates a 2-fold change threshold, whereas a red dashed line indicates a 4-fold change threshold. All treatments included a total of six replicates (*n* = 6).

**Figure 9:**
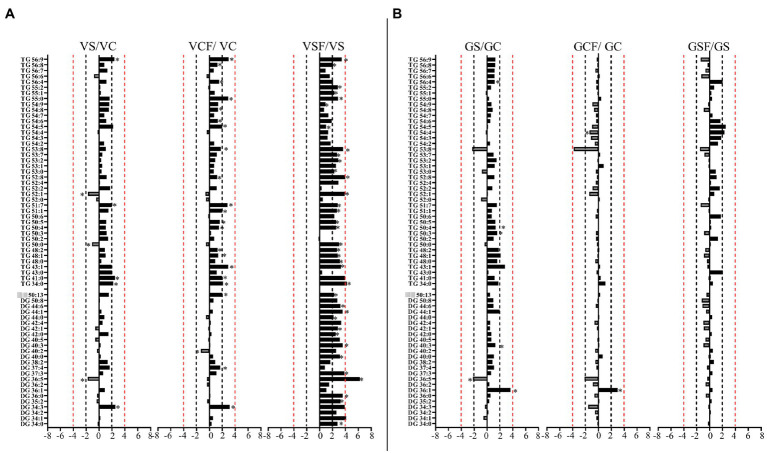
Logarithmic ratios of representative diacylglycerol (DG) and triacylglycerol (TG) content in roots of barley cv. Vlamingh (V) given as **(A)** and cv. Gairdner (G) given as **(B)**, showing three comparisons—salt grown (200 mM NaCl) vs. control grown (*VS*/VC and GS/GC); control with fungus vs. control grown (VCF/VC and GCF/GC); salt and fungus grown vs. salt grown (VSF/*VS* and GSF/GS). An asterisk (^*^) indicates that the values are significantly different (*p* < 0.05, FDR). A black dashed line indicates a 2-fold change threshold, whereas a red dashed line indicates a 4-fold change threshold. All treatments included a total of six replicates (*n* = 6).

Salt treated caused more significant changes in Vlamingh than in Gairdner. When salt treated roots were compared to untreated control roots, nine lipids from GP (Figures 7A, 8A) and five from GL class (Figure 9A) increased with significant changes greater than +1.5-fold in Vlamingh and eight lipids from GP and five from GL class showed significant changes greater than +1.5-fold in Gairdner. Salt stress significantly reduced nine lipid species in Vlamingh from the GP class with changes ranging between −1.5-fold and − 2.1-fold reduction, six of these were lysophospholipids (LPC 18:2, LPC 20:4, LPC 22:n, LPC 25:0, LPC 28:1 and LPE 20:2; Figures 7A, 8A). Only one lipid (DG 36:5) was significantly reduced in Gairdner with a − 2.14-fold decrease after salt stress (Figure 9B).

In Vlamingh, fungal inoculation (treatment *CF*) resulted in a statistically significant rise in a much larger number of lipids than in Gairdner. In inoculated Vlamingh seedlings, 25 lipid species from GP class showing fold change between +1.5-fold and + 2.76-fold and 22 lipids from the GL class with fold changes greater than +1.5-fold when compared to uninoculated Vlamingh (Figures 7A, 8A). However only DG 36:1 (+2.82-fold) increased in inoculated Gairdner (Figure 9A). Six lipids from the GP and GL classes were reduced in inoculated Gairdner (Figures 7B, 8B, 9B), whereas only four lipids were reduced in Vlamingh after inoculation (Figures 7A, 8A, 9A).

When fungus-inoculated roots were treated with salt (treatment SF), Vlamingh showed more alterations than Gairdner when compared to uninoculated plants treated with salt (S). After salt treatment, in inoculated roots of Vlamingh, 18 PCs and eight LPCs were increased with fold changes between +1.5 and + 6.5-fold (Figure 7A). However, in inoculated roots of Gairdner, four PCs and one LPC were reduced after salt stress (Figure 7B).

Like PCs, 21 PEs with fold changes ranging from +1.5-fold to +5.41-fold, and three LPEs with fold changes between +1.5-fold and + 6.22-fold were significantly increased in inoculated roots of Vlamingh under saline conditions. In addition to these, inoculated roots of Vlamingh also had five PGs (33:n and 36:n family), two PIs (33:n) and six PSs (34:6, 38:1, 38:4, 40:1, 42:4 and 42:5) that were increased under saline conditions (Figure 8A) with fold changes between +1.5 and + 4.16. However, similar to PCs, in inoculated roots of Gairdner PEs and LPEs were reduced following salt stress (Figure 8B) except LPE (23:0) which increased after salt stress.

The inoculated roots of Vlamingh showed profound changes in GL lipid species following salt stress with 13 DGs (from 36:n to 44:0) and 23 TG s increased with fold changes between +1.5 and + 8.74-fold (Figure 9A). In inoculated roots of Gairdner treated with salt, no major changes were observed in the GL class.

## Discussion

This study demonstrated that *T. harzianum* T-22 improved the growth of two barley genotypes grown in control and saline conditions. Although this study does not directly compare the responses of the two genotypes to fungal inoculation and salt stress, our results demonstrate that metabolites produced by the two genotypes are different and we make some general comments about these differences in the discussion below. The physical changes caused by inoculation and salt treatment of the two barley genotypes were identified by measuring a range of parameters. There was a general pattern that inoculation with fungus improved most of these measures compared to uninoculated plants, but significant differences caused by inoculation were only seen after salt treatment. While uninoculated plants were severely affected after salt treatment, in inoculated plants there was little effect of salt on SRL, shoot and root dry weight, shoot chlorophyll content and root: shoot ratio; they grew as well as (or better than) uninoculated plants grown without salt. The sensitive cultivar Gairdner was more severely affected by salt than the tolerant cultivar Vlamingh but at least for SRL the improvement in growth in saline conditions was greater in Gairdner, perhaps because the effect of the salt treatment was greater. However, in general, inoculated Vlamingh grew better in saline conditions than inoculated Gairdner when compared to uninoculated control plants.

Salt stress decreased root to shoot ratio in the tolerant cultivar Vlamingh. There was a slight reduction in seminal root length and overall root biomass in salt stressed seedlings as compared to control seedlings in Vlamingh. This approach is likely to reduce the likelihood of the new roots being damaged in saline conditions. In contrast, root to shoot ratio in the sensitive cultivar Gairdner under saline conditions was similar to control seedlings with above ground biomass reduced while below ground biomass (determined by root and shoot fresh and dry weight measurements in salt compared to no salt treatments) was maintained. This property encourages accumulation of harmful ions in roots, decreasing their transfer to shoots. In saline environments this is a typical mechanism of plant resilience (Cassaniti et al., 2009, 2012). Inoculation with fungus under saline conditions increased root to shoot ratio in Vlamingh but decreased it in Gairdner, suggesting the fungus enhances the formation of nodal and lateral roots in the sensitive genotype after inoculation under salt stress. This could be an adaptive mechanism of the sensitive genotype for better nutrient and water absorption needed for resistance against salt stress.

Salinity reduced the chlorophyll content in both genotypes in this study with greater reduction in the sensitive genotype, Gairdner. Similar results were reported by Khan et al. (2009) where a significant decrease in chlorophyll content of sensitive genotypes of wheat was seen in comparison to tolerant genotypes. Roy and Srivastava (2000) and Ashraf and Foolad (2005) reported that reduction in chlorophyll contents in rice and wheat under saline conditions is associated with disturbances in membrane stability. Inoculation with fungus under control and saline conditions resulted in higher chlorophyll content in both genotypes suggesting increased photosynthetic capacity imparted by the fungus in these plants. It can also be presumed that fungal association with plants improves the plant’s capacity for gas exchange and the efficiency of photosynthetic electron transport in inoculated plants as shown by the maintenance of an efficient photochemistry. Similar results were observed in soybean plants (Khan et al., 2011a) and Bermuda grass (Xie et al., 2017) inoculated with an endophyte under saline conditions.

### Protective Adaption to Salt Stress Due to Osmolytes Changes Caused by the Fungus in Roots

Under control and saline conditions, a global analysis of the GC–MS dataset using PCA and HCA revealed significant osmolyte variations between the two inoculated and uninoculated barley genotypes. After salt stress, Gairdner showed more significant changes in sugars and amino acids than Vlamingh. These metabolites are typically thought of as suitable solutes that help with osmotic adjustment and protect membranes from reactive oxygen species damage (ROS; Wang et al., 2008). Higher contents of these metabolites in Gairdner suggest a greater need for stabilising the cell membrane and protoplast in the more sensitive genotype. Similar results were shown by Cao et al. (2017) where barley genotypes with different levels of salt tolerance changed their sugar metabolism in different ways after salinity stress.

Proline is the only amino acid which accumulated at higher levels in both genotypes. Similar substantial increases in proline were reported by Chen et al., (2007); Widodo Patterson et al. (2009); and Wu et al. (2013). The physiological significance of proline accumulation is controversial, while some experts believe it is an indication of stress (Hernandez et al., 2000; Rai et al., 2003), others suggest that it acts as a solute for intercellular osmotic adjustment when found in high concentrations (Silveira et al., 2003; Kaur and Asthir, 2015). Proline can act as an antioxidant and operate as a molecular chaperone to protect the structure of biological macromolecules during dehydration, providing tolerance to environmental challenges in addition to acting as an osmoprotectant (Ashraf and Foolad, 2007; Tang et al., 2015).

After inoculation with the fungus under control conditions there were more changes in organic acids in inoculated Vlamingh than in Gairdner. The fungus may promote nutrient uptake in Vlamingh by inducing secretion of organic acids to make nutrients in the soil more available to the plant. However, this hypothesis requires further investigation.

Inoculation with the fungus under control conditions resulted in increases in amino acids in both genotypes. Amino acids are the building blocks of proteins and Akladious and Abbas (2012) showed that the protein content is influenced by the application of plant growth promoting microbes, including fungi (Akladious and Abbas, 2012). Thus, we can speculate that the fungus may enhance the N accumulation in both barley genotypes and subsequently the amino acid composition. Similar results were obtained by Mishra and Nautiyal (2018) where *Trichoderma* spp. increased the relative abundance of amino acids in chickpeas.

Under saline conditions, the fungus appears to activate different mechanisms to alleviate the effects of salt stress in the two genotypes. In inoculated Vlamingh, sugars are accumulated after salt stress. However, in Gairdner, sugars are decreased and thus it can be presumed that the fungus helps in translocation of osmolytes to the leaves, to mitigate the effects of salt stress by reducing the levels of sugars under saline conditions. This is also supported by our physiological results where shoot dry weight and chlorophyll content was increased in inoculated Gairdner seedlings following salt stress, though further confirmation to measure shoot metabolite content is needed to validate these results.

As for sugars, inoculation with the fungus caused a significant increase in the contents of amino acids in Vlamingh and a decrease in Gairdner. This suggests that in Vlamingh the fungus transforms nutrients to make them more bioavailable, which in turn promotes soil circulation and reduces the requirement for nitrogen fertiliser (Harman, 2011). Similar results were reported by Maeda et al. (2015) showing the ability of *Trichoderma* spp. to decompose nitrogen compounds into available nitrogen and release less NO_2_. In Gairdner where fungal inoculation causes a considerable decrease in amino acid levels, the fungus may improve the sensitive genotype’s ability to endure salt stress by improving metabolism of amino acids and so contributing to plant growth. This is in line with recent field studies, in which *Aspergillus aculeatus* inoculated roots of ryegrass plants had lower amino acid contents after salt stress (Li et al., 2017).

Under salt stress, the salt sensitive genotype Gairdner had significant increases in organic acid levels but in the same conditions fungus inoculated roots had much lower levels of organic acids. Very little information on the role of fungus in changing organic acid profiles in salt stressed plants is available in the literature and thus, this calls for further investigation.

### Changes in the Lipid Profiles in Vlamingh and Gairdner Under Three Treatments

To elucidate the role of lipids in adaptive mechanisms of inoculated and uninoculated roots after short-term salt stress, untargeted lipidome analyses were performed on roots of both genotypes. The untargeted data suggests that the genetic differences between the genotypes had a strong impact on the root lipidome with no strong pattern of changes resulting from salt treatment or fungal inoculation. In both genotypes the biggest changes in lipids resulted from inoculation with fungus rather than the salt treatment.

Several lipid classes, including PC, PS, PE, and TG, show patterns of change in Vlamingh that could be linked to salt tolerance. On the other hand, few or no changes were observed in lipid classes in Gairdner and thus it can be assumed that other macromolecules are responsible for increased biomass seen in salt treated inoculated plants compared to uninoculated plants.

Salt stress increased PC in Vlamingh suggesting the net production of PCs from DG through DP-choline:diacylglycerol cholinephosphotransferase (CPT) as DGs were decreased after salt stress. Similar results were observed when this study was performed on agar media as explained in Gupta et al. (2021). However, increases in PCs in inoculated Vlamingh under control conditions can be attributed to the ability of the fungus to successfully penetrate into the roots resulting in increased membrane lipids (Chukwuma et al., 2020). This is further supported by our findings with increased biomass in Vlamingh post inoculation resulting in increased accumulation of membrane lipids. Similar results were shown by Wei et al. (2016) where *Ericoid* mycorrhizal (ERM) fungi *Oidiodendron maius* var. increased expression of genes involved in glycerophospholipid metabolism in inoculated roots of *Rhododendron fortunei* Lindl although the actual levels of these lipids were not determined. The same PCs increased by salt stress were further increased after fungal inoculation of Vlamingh. This could be due to the role of the fungus in maintaining membrane structure and function as a response to salt stress in the tolerant genotype Vlamingh. Several studies support our findings where *Trichoderma* spp. increased tolerance to salt stress by improved root growth and enhanced nutrient uptake. In contrast, Gairdner either maintained or showed decreased levels of PCs in both control and saline conditions with and without inoculation. Here, it can be suggested that Vlamingh is already adapted to tolerate salt but the fungus gave Gardiner the ability to react to salt stress using a similar mechanism. This adaptive mechanism has been demonstrated in salt-sensitive plants such as maize, oats and wheat as well as salt-sensitive barley genotypes (Norberg et al., 1991; Magdy et al., 1994; Salama et al., 2007; Salama and Mansour, 2015).

Plants can synthesise distinct molecular types of diacylglycerol (DG), the immediate precursor of TG, *via* at least two metabolic pathways: (Food and Agriculture Organization of the United Nations, 2018) *de novo* DG synthesis and [United Nations Department of Economic Social Affairs (UN DESA), 2019] conversion of the membrane lipid phosphatidylcholine (PC) to DG. Our results are similar to those of Gupta et al. (2021) and suggest that Vlamingh and Gairdner use different pathways to synthesise DGs in response to salt stress.

In Vlamingh, the results for the production of phosphatidylcholines (PCs), diacylglycerols (DGs) and TGs in response to the different treatments support the idea that for inoculated Vlamingh grown in control conditions, DGs are synthesised *de novo* and incorporated into TGs *via* the Kennedy pathway (Gupta et al., 2021). However, when subjected to salt stress (inoculated or uninoculated) DGs are produced by conversion of the membrane lipid PC to DG (Gupta et al., 2021). This lipid remodelling involves fatty acids being channelled from constitutive membrane lipids to TGs. Furthermore, because TGs mostly include polyunsaturated fatty acids, the fungus may facilitate the release of polyunsaturated fatty acids from structural lipids and their use for transitory TG assembly during membrane remodelling. Similar results were observed by Mueller et al. (2015) where salt stress triggered accumulation of TGs in Arabidopsis seedlings.

On the other hand, as there was no significant increase in PC species for any treatment in Gairdner, our data suggest that DG/TG synthesis proceeds through direct use of FA synthesis products exported from the plastid for *de novo* DG/TG synthesis (DG1) *via* the Kennedy pathway (Weiss and Kennedy, 1956; Weiss et al., 1960), as seen by Gupta et al. (2021). Further investigation of a wider range of lipid classes such as phosphatic acids (PA), phosphatidylinositols (PI) and PG, especially utilising negative ionisation mode analyses may identify changes that were not detected in this study.

Since this study was one of the first to investigate the changes in lipidome of roots subjected to control and saline conditions with and without fungus, many questions remain unanswered and therefore, this calls for future studies in the field to further explore the potential changes in lipids of both genotypes brought about by the inoculation with fungus.

The increased biomass in inoculated Vlamingh after salt stress seen here can be attributed to the increased levels of metabolites and lipids caused by fungal inoculation providing protection from salt damage and so improving plant growth. However, it is not clear whether increased physiological capacity and higher biomass in inoculated Gairdner is due to the metabolites identified here or other macromolecules not identified in this study. As a result, more research is needed to confirm the relationships we have discovered between enhanced growth, modifications in the lipid and metabolite profile, and salt tolerance.

Gupta et al. (2021) studied the changes in the metabolome and lipidome of the same barley genotypes used in this study, but grown on agar media. While physiological results show that salt treatment reduces root and shoot length and weight, as well as chlorophyll content in both genotypes in this study, fungal inoculation restores growth to better than or close to normal, confirming the fungus’ positive role in helping plants cope with the negative effects of salt stress. As in Gupta et al. (2021), the *T. harzianum* fungus modified the metabolome and lipidome in each cultivar in a specific manner by enhancing different metabolic pathways in the different genotypes. However, fewer metabolite changes, also with less amplitude, were observed when plants were grown on agar media as compared to this study when plants were grown in soil. Several classes of lipid species (PG-phosphatidylglycerol, PI-phosphatidylinositol, PS-phosphatidylserine) were detected with different response patterns when plants were grown in soil as compared to agar media. Our results demonstrate the importance of using conditions as close as possible to a field environment (i.e. plants grown in soil) when studying the molecular responses of roots to soil salinity stress. It also demonstrates the ability of the fungus to manipulate plant metabolism in different ways depending on the plant’s innate ability to deal with saline conditions.

## Conclusion

This study investigated the changes in levels of metabolites and lipids in two barley genotypes, Vlamingh and Gairdner, inoculated with fungal endophyte *T. harzianum* strain T-22 and grown in soil under control and saline conditions. To identify how each genotype responds to fungal inoculation and salt stress and how inoculation improves tolerance, several physiological parameters such as root and shoot length and weight and chlorophyll content were measured. Further various orthogonal approaches were combined to compare and contrast the lipid and metabolite profiles in the different treatments using GC–MS and LC-QqTOF-MS. Use of these approaches as exploratory and qualitative techniques provide evidence of the molecular adaptations brought about by changes in metabolites and lipids after fungal inoculation of barley roots under saline soil conditions. These findings pave the way for further investigation of the processes by which fungal inoculation alters plant metabolism. The interesting differences in the metabolic response of the two genotypes to fungal inoculation can be explored in further studies. Analysis of larger field trials to confirm the role of fungus in several cereal crops is required. However, these initial results confirm the positive role of the endophyte in imparting salt tolerance, especially to the less salt-tolerant barley variety, Gairdner.

## Data Availability Statement

The original contributions presented in the study are included in the article/Supplementary Material; further inquiries can be directed to the corresponding author.

## Author Contributions

SG, SN, PS, and UR designed the research. SG performed the experiments with the help of PS and SN. SG analysed the data. SG and PS wrote the manuscript with inputs from other authors. All authors contributed to the article and approved the submitted version.

## Funding

This project was supported through a Melbourne Research Scholarship to SG and University of Melbourne internal funds to UR.

## Conflict of Interest

The authors declare that the research was conducted in the absence of any commercial or financial relationships that could be construed as a potential conflict of interest.

## Publisher’s Note

All claims expressed in this article are solely those of the authors and do not necessarily represent those of their affiliated organizations, or those of the publisher, the editors and the reviewers. Any product that may be evaluated in this article, or claim that may be made by its manufacturer, is not guaranteed or endorsed by the publisher.
