# Supplementary material for: Biochemical Changes in Two Barley Genotypes Inoculated With a Beneficial Fungus Trichoderma harzianum Rifai T-22 Grown in Saline Soil
Source: Front Plant Sci. 2022 Aug 2;13:908853. doi: 10.3389/fpls.2022.908853 (PMC9379338; doi:10.3389/fpls.2022.908853)
Supplement: Supplementary file 1 [file Data_Sheet_1.PDF]

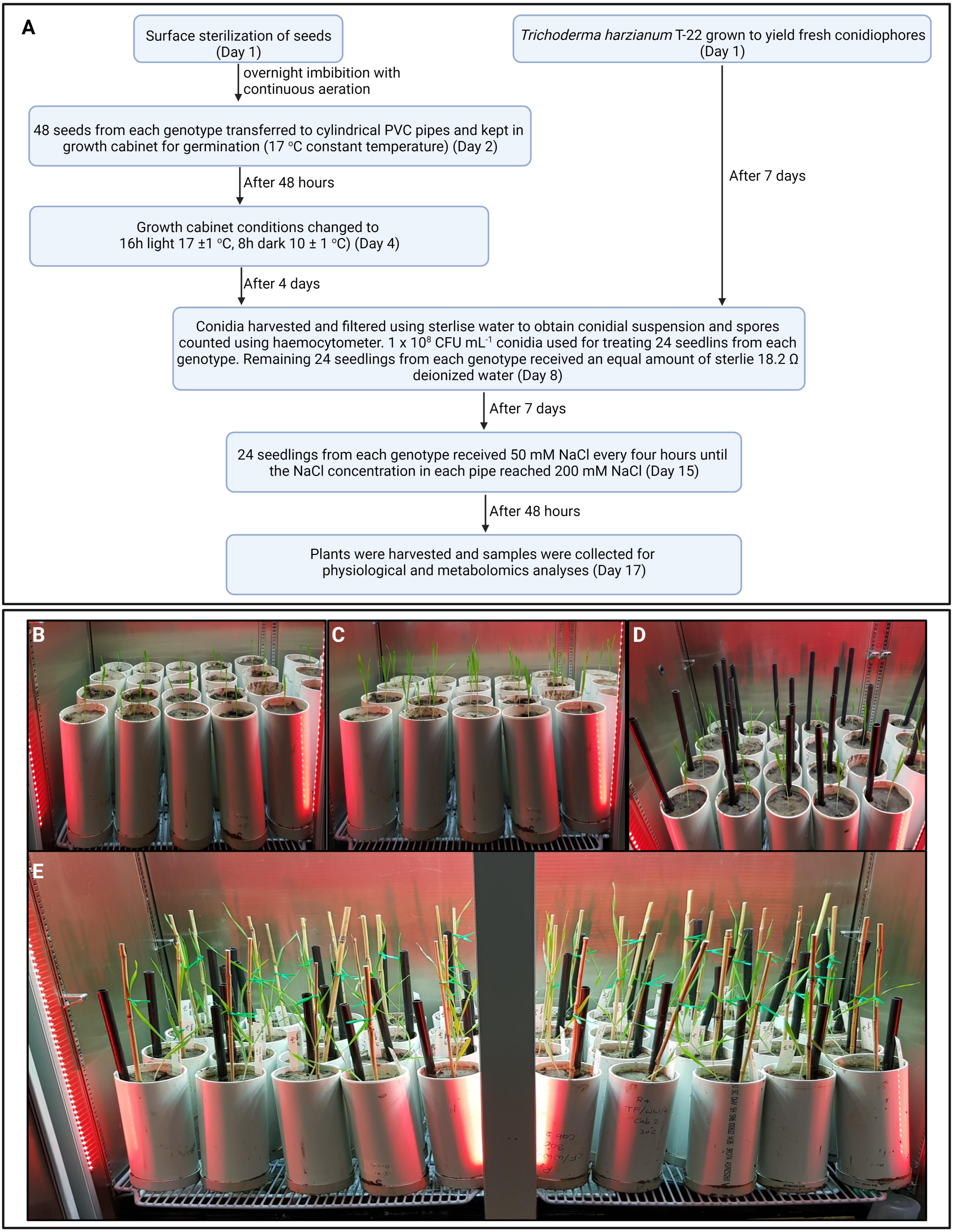

Supplementary Figure 1. Flowchart for the experimental setup for plant growth conditions and *Trichoderma harzianum* T-22 inoculation (A) and images enhibiting plant growth for second experiment of the study. Images are captured at, B, 3 days; C, 6 days; D, 9 days; and E, 18 days from sowing

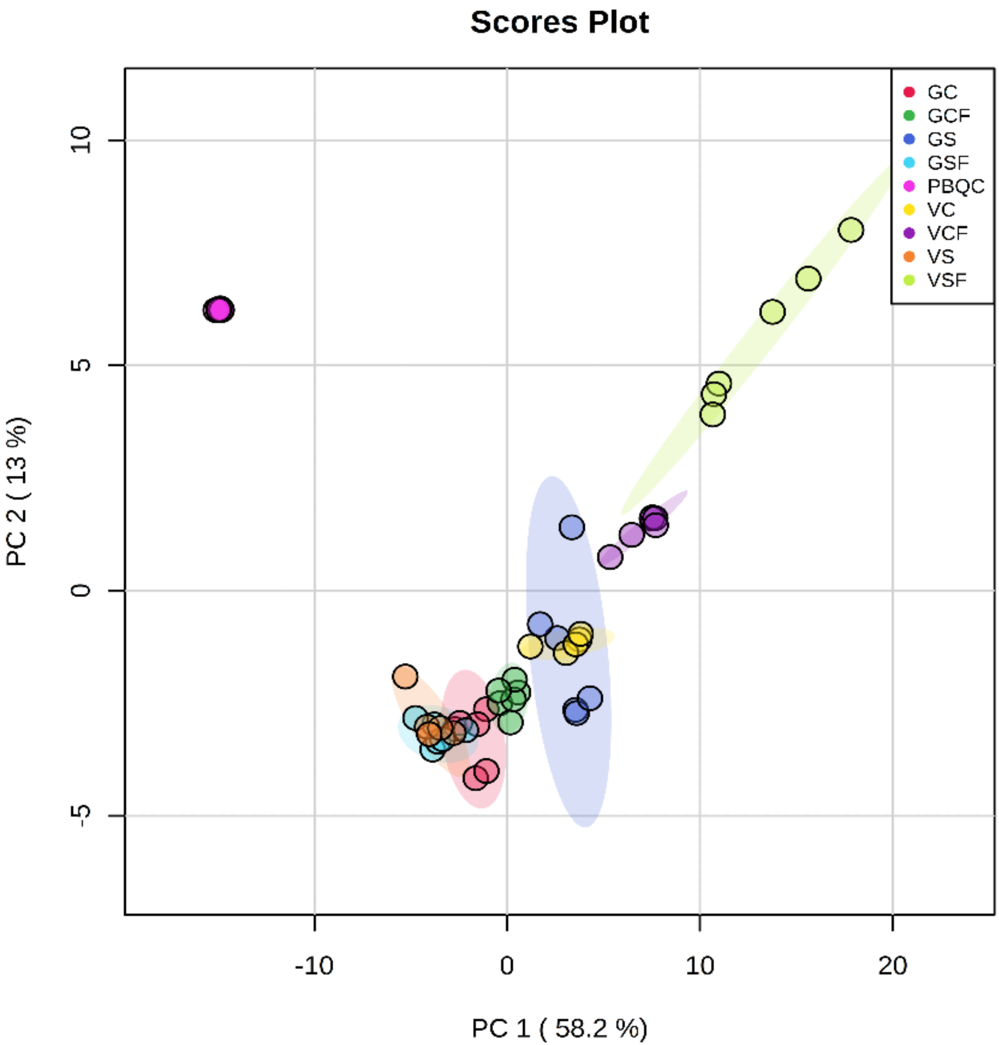

Supplementary Figure 2. Principal Component Analysis (PCA) score plot for two barley genotypes - cv. Vlamingh and cv. Gairdner from four treatments - control, control + fungus, salt, and salt+ fungus - and quality control samples. Abbreviations: G- cv. Gairdner, V- cv. Vlamingh, C- control treated roots, S- Salt treated roots (200 mM NaCl), CF- control + fungus, SF- salt + fungus, PBQC- pooled biological quality control samples

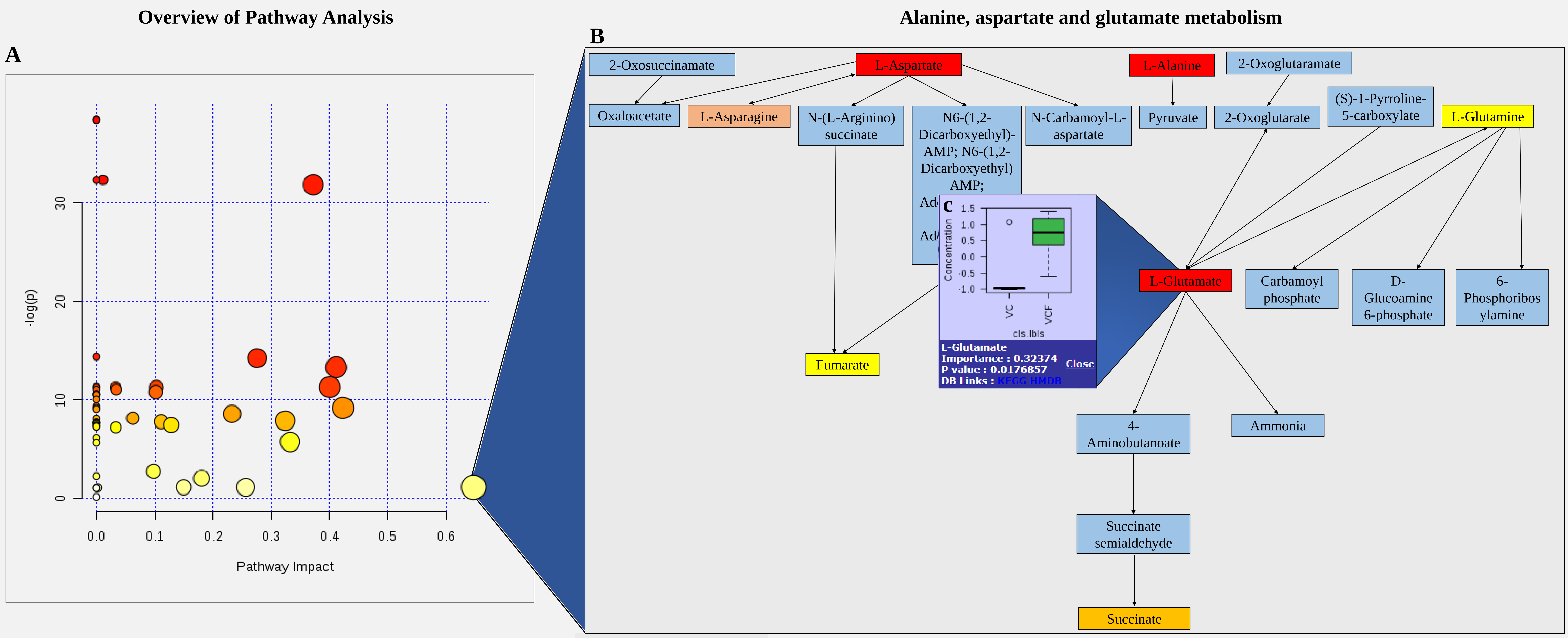

Supplementary Figure 3. Overview of pathway analysis, corresponding pathway view and significantly changed compound - L-glutamate in cv. Vlamingh treatment control + fungus (CF) compared to control (C). (A): metabolome view- pathway impact is given on X-axis and log (p value) on Y-axis. The sum of the important measures of the matched metabolites is normalised by the sum of the importance measures of all metabolites in each pathway to compute the pathway impact. The colour of a node is determined by its p value, and the radius of a node is determined by its pathway impact values. Red colour indicates the most significant pathway and light-yellow colour indicates least significant pathway. (B): Corresponding pathway view for node colour given in yellow. Alanine, aspartate and glutamate metabolism is highlighted by pointing and clicking on hyperlinked nodes (yellow colour above in ‘A’). (C): Single metabolite node. L-glutamate is highlighted as an example to compare its concentration between CF and C in cv. Vlamingh.

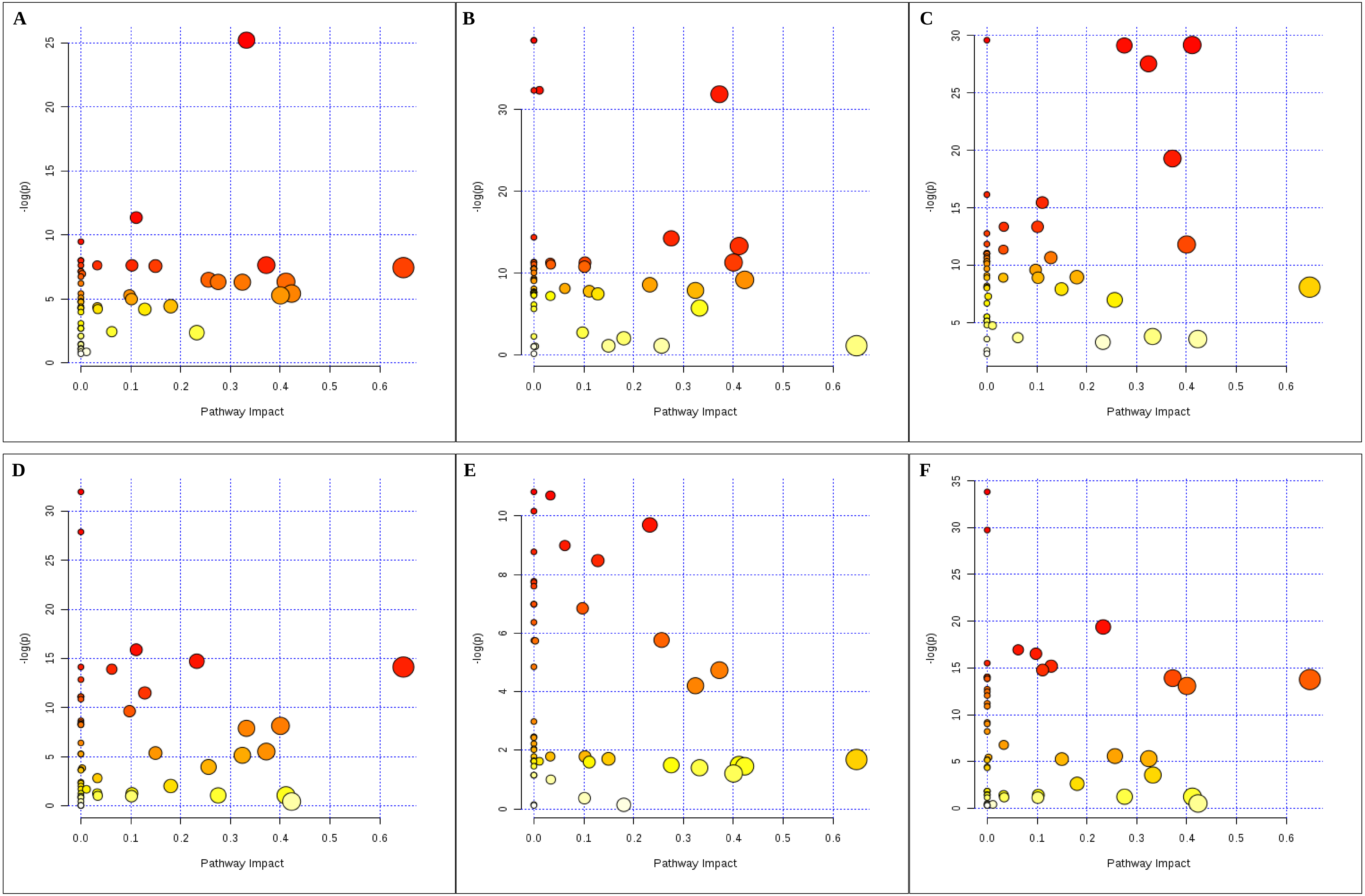

Supplementary Figure 4. Metabolome view for all identified metabolites of barley cv. Vlamingh (V) and cv. Gairdner (G) of salt grown compared to control grown (a. VS/VC and d. GS/GC); control with fungus compared to control grown (b. VCF/VC and e. GCF/GC); and salt with fungus compared to salt grown (c. VSF/VS and f. GSF/GS). All matching pathways are grouped by p values on the Y-axis and pathway impact values on the X-axis in the metabolome view. The colour of the nodes is decided by their p value, and the radius of the nodes is determined by their pathways impact values.

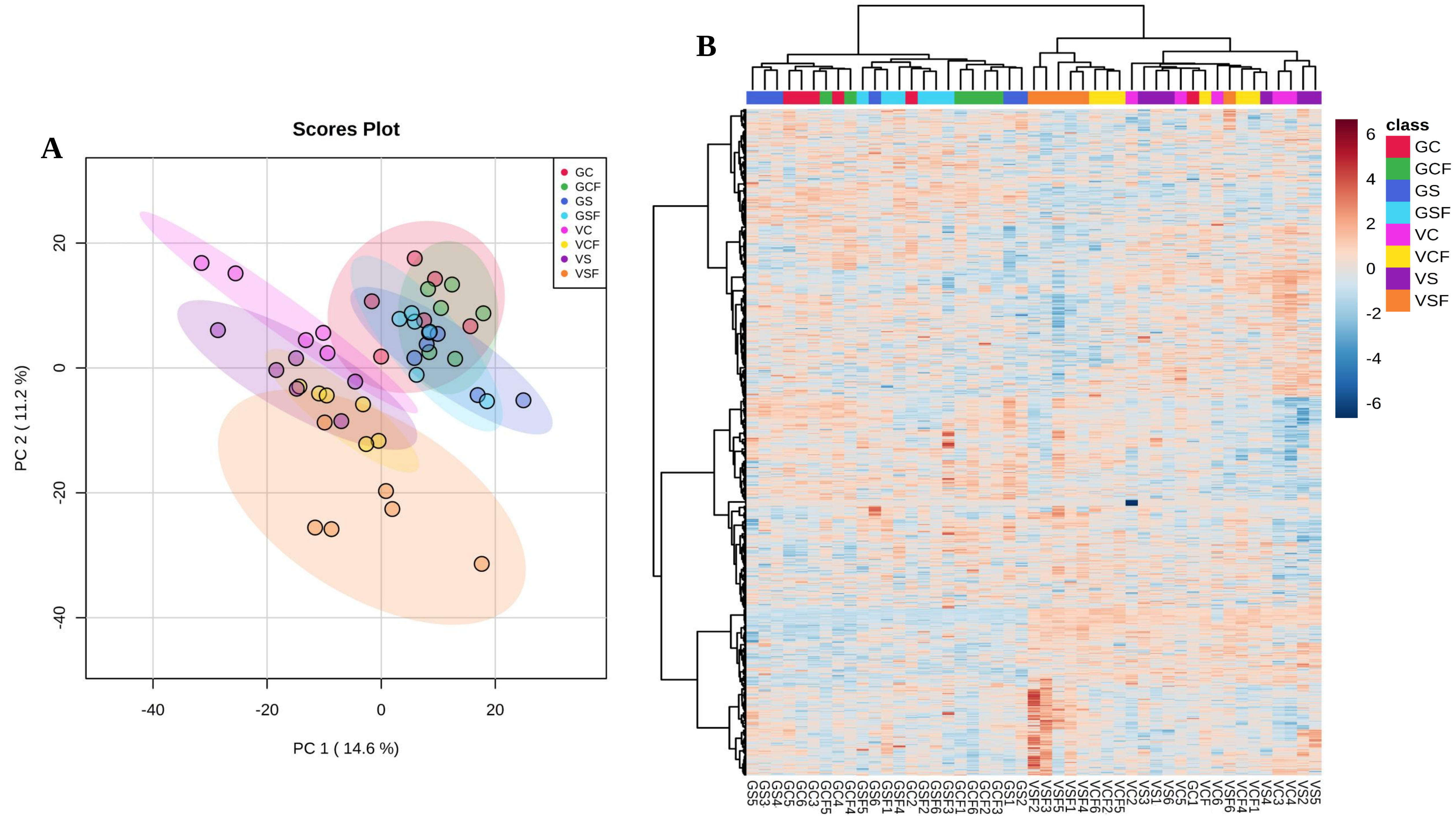

Supplementary Figure 5. Principle Component Analysis (PCA) and heatmap of the mass features (m/z_RT) extracted from lipid profiles from roots of two barley genotypes (cv. Vlamingh and cv. Gairdner) grown under control and saline conditions, with and without fungus. 5a- PCA showing scores plot for detected m/z_RT for Control- C, Control + fungus- CF, Salt- S and Salt + fungus- SF in cv. Vlamingh and cv. Gairdner; 5b- heatmap generated from hierarchical cluster analysis (HCA) of all detected m/z_RT features in cv. Vlamingh and cv. Gairdner. The sample groups are indicated by the labels at the bottom.

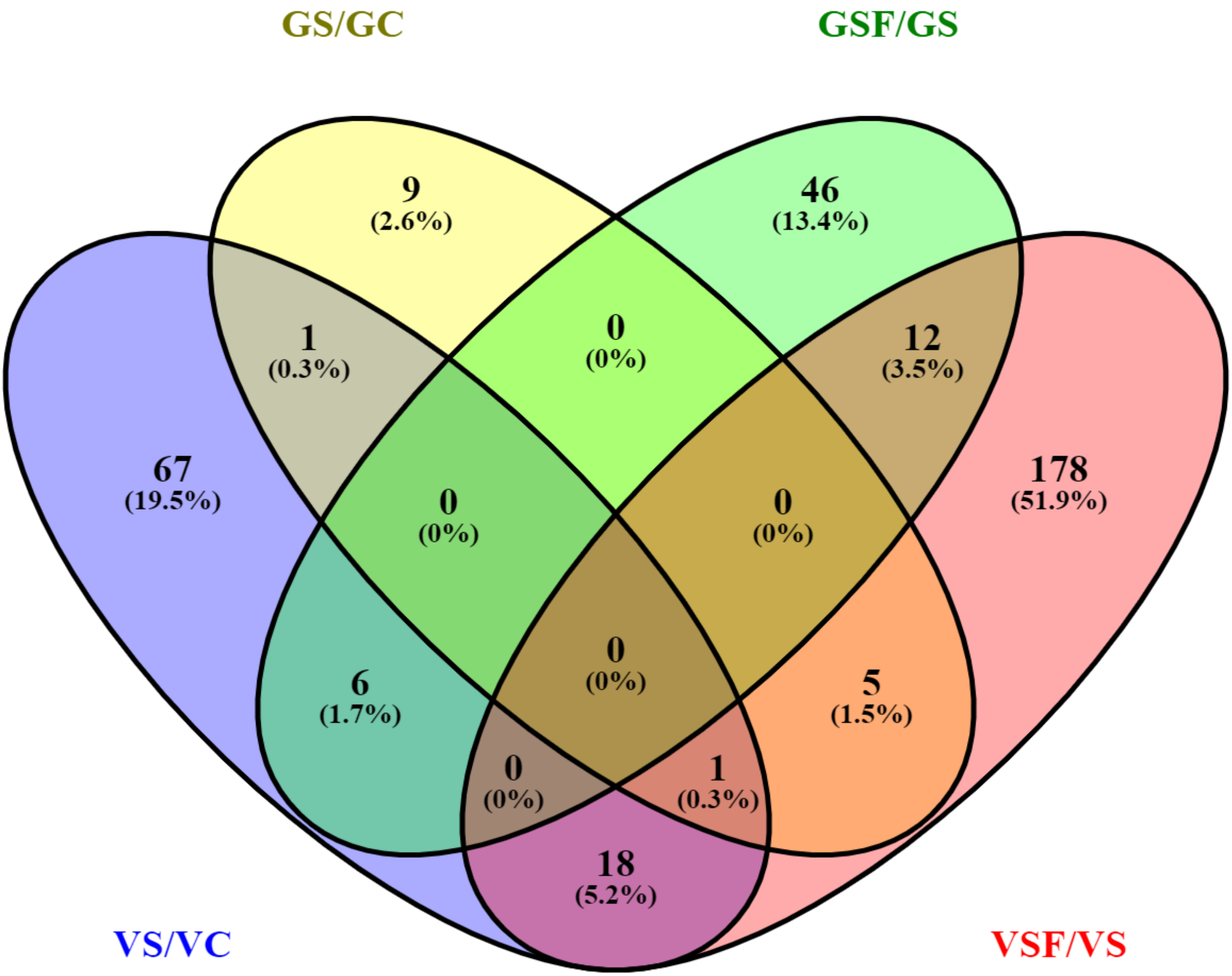

Supplementary Figure 6. The number of significantly altered characteristics discovered by one-way ANOVA and post-hoc analysis of lipid profiles from roots of two barley genotypes under salt stress and with and without fungal inoculation is summarised in a Venn diagram. Regardless of their directionality (upregulation and downregulation), the number represents significantly altered features. Numbers appearing in overlapped sections are common between treatments. Four analysis were performed- the cv. Vlamingh salt (VS) was compared to the cv. Vlamingh control (VC), the cv. Vlamingh salt and fungus (VSF) was compared to the cv. Vlamingh salt (VS), the cv. Gairdner salt (GS) was compared to the cv. Gairdner control (GC), and the cv. Gairdner salt and fungus (GSF) was compared to the cv. Gairdner salt (GS).

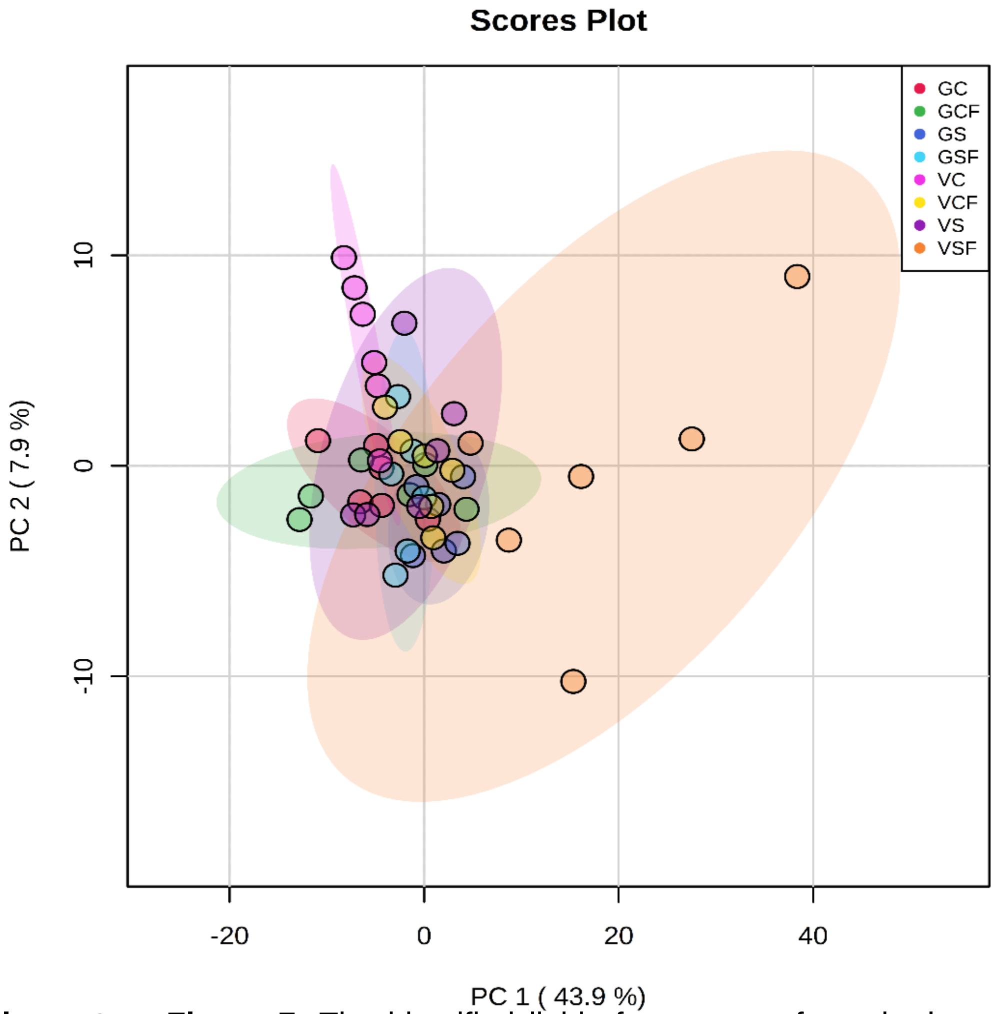

Supplementary Figure 7. The identified lipids from roots of two barley genotypes grown under control and saline conditions, with and without fungus, were subjected to principle component analysis (PCA). VC stands for control treated cv. Vlamingh; VCF stands for control and fungus treated cv. Vlamingh; VS stands for salt treated cv. Vlamingh; VSF stands for salt and fungus treated cv. Vlamingh; GC stands for control treated cv. Gairdner; GCF stands for control and fungus treated cv. Gairdner; GS stands for salt treated cv. Gairdner; GSF stands for salt and fungus treated cv. Gairdner. VSF is distinguished from the other groups through PC 1 versus PC 2. All treatments included a total of six replicates (n = 6). PC2 was unable to distinguish between treatments or cultivars.

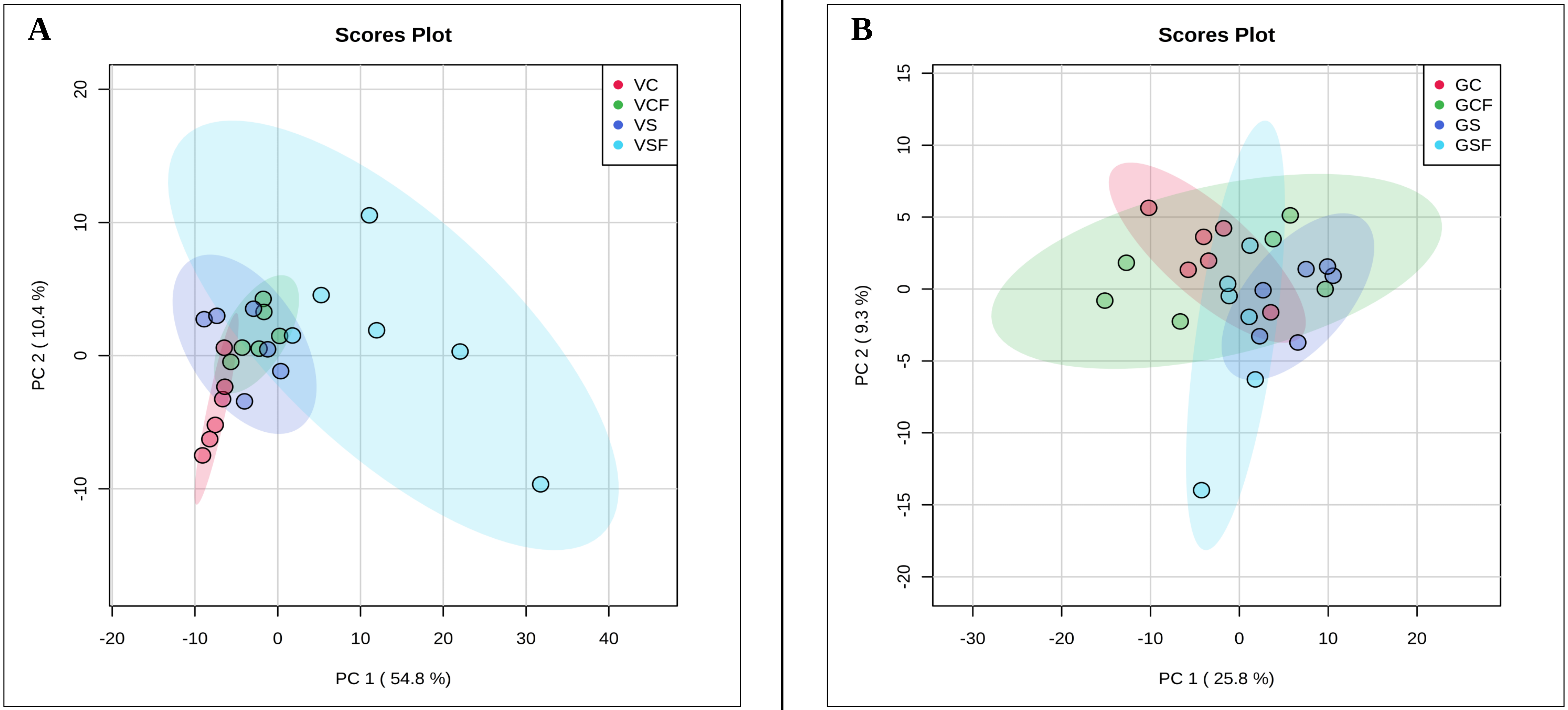

Supplementary Figure 8. The identified lipids from roots of two barley genotypes grown under control and saline conditions, with and without fungus, were subjected to principle component analysis (PCA). VC- control treated cv. Vlamingh; VS- salt treated cv. Vlamingh; VCF- control and fungus t reated cv. Vlamingh; GC- control treated cv. Gairdner; GS- salt treated cv. Gairdner; GCF- control and fungus treated cv. Gairdner, GSF- salt and fungus treated cv. Gairdner. The separation between SF and other groups in cv. Vlamingh can be seen in PC 1 versus PC 2 (A). In cv. Gairdner, PCA failed to demonstrate any clear separation (B). All treatments included a total of six replicates (n = 6).

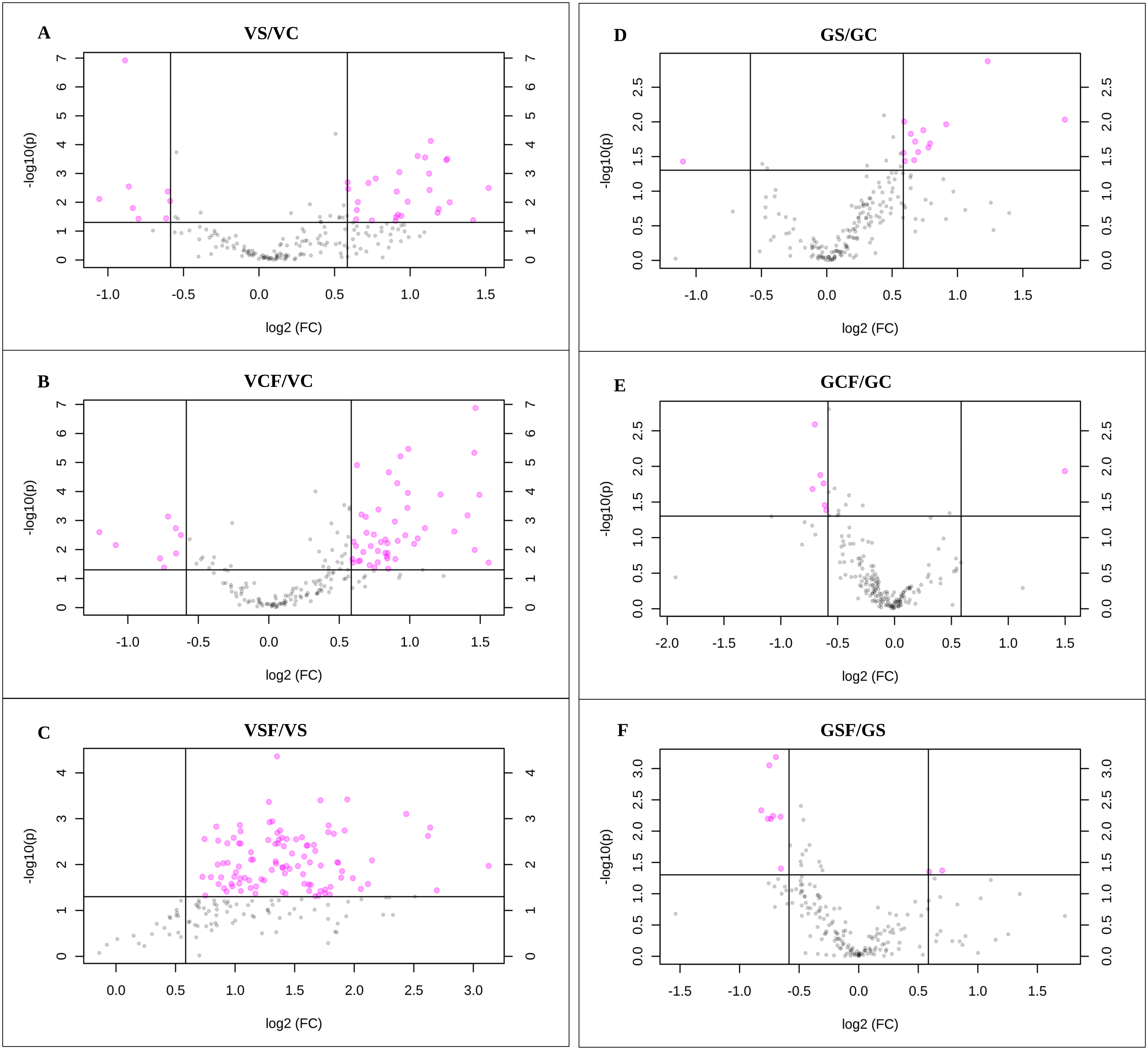

Supplementary Figure 9. Volcano plots of significantly affected lipids in the comparison from roots of two barley genotypes grown under control and saline conditions, with and without fungus. A- Vlamingh (V) of salt grown compared to control grown (VS/VC); B- Vlamingh (V) of control with fungus compared to control grown (VCF/VC); C- Vlamingh (V) of salt with fungus compared to salt grown (VSF/VS); D- Gairdner (G) of salt grown compared to control grown (GS/GC); E- Gairdner of control with fungus compared to control grown (GCF/GC); F- Gairdner of salt with fungus compared to salt grown (GSF/GS).
